# Tetra-glucopyranosyl Diterpene *ent*-Kaur-16-en-19-oic Acid and *ent*-13(*S*)-Hydroxyatisenoic Acid Derivatives from a Commercial Extract of *Stevia rebaudiana* (Bertoni) Bertoni

**DOI:** 10.3390/molecules23123328

**Published:** 2018-12-15

**Authors:** Wilmer H. Perera, Ion Ghiviriga, Douglas L. Rodenburg, Kamilla Alves, Frank T. Wiggers, Charles D. Hufford, Frank R. Fronczek, Mohamed A. Ibrahim, Ilias Muhammad, Bharathi Avula, Ikhlas A. Khan, James D. McChesney

**Affiliations:** 1 Ironstone Separations, Inc., Etta, Oxford, MS 38627, USA; wilmer.perera@gmail.com (W.H.P.); douglasrodenburg@yahoo.com (D.L.R.); Kamilla_07@yahoo.com (K.A.); 2 ORISE Fellow-Agricultural Research Service, Natural Product Utilization Research Unit, U.S. Department of Agriculture, University of Mississippi, Oxford, MS 38677, USA; 3 Department of Chemistry, University of Florida, Gainesville, FL 32611, USA; ion@chem.ufl.edu; 4 National Center for Natural Products Research, University of Mississippi, Oxford, MS 38677, USA; fwiggers@olemiss.edu (F.T.W.); mmibrahi@olemiss.edu (M.A.I.); milias@olemiss.edu (I.M.); bavula@olemiss.edu (B.A.); ikhan@olemiss.edu (I.A.K.); 5 Department of Chemistry, Louisiana State University, Baton Rouge, LA 70803, USA; ffroncz@lsu.edu; 6 Chemistry of Natural Compounds Department, Pharmaceutical and Drug Industries Division, National Research Centre, Dokki, Giza 12622, Egypt

**Keywords:** *Stevia rebaudiana*, diterpene glycosides, rebaudioside A isomers, 13(*S*)-hydroxyatisenoic acid derivative, iso-stevioside X-ray structure

## Abstract

*Stevia rebaudiana* and its diterpene glycosides are one of the main focuses of food companies interested in developing novel zero calorie sugar substitutes since the recognition of steviol glycosides as Generally Recognized as Safe (GRAS) by the United States Food and Drug Administration. Rebaudioside A, one of the major steviol glycosides of the leaves is more than 200 times sweeter than sucrose. However, its lingering aftertaste makes it less attractive as a table-top sweetener, despite its human health benefits. Herein, we report the purification of two novel tetra-glucopyranosyl diterpene glycosides **1** and **3** (rebaudioside A isomers) from a commercial *Stevia rebaudiana* leaf extract compounds, their saponification products compounds **2** and **4**, together with three known compounds isolated in gram quantities. Compound **1** was determined to be 13-[(2-*O*-β-d-glucopyranosyl*-*6-*O*-β-d-glucopyranosyl-β-d-glucopyranosyl) oxy]*ent*-kaur-16-en-19-oic acid-β-d-glucopyranosy ester (rebaudioside Z), whereas compound **3** was found to be 13-[(2-*O*-β-d-glucopyranosyl*-*3-*O*-β-d-glucopyranosyl-β-d-glucopyranosyl) oxy]*ent*-hydroxyatis-16-en-19-oic acid -β-d-glucopyranosy ester. Two new tetracyclic derivatives with no sugar at position C-19 were prepared from rebaudiosides **1** and **3** under mild alkaline hydrolysis to afford compounds **2** 13-[(2-*O*-β-d-glucopyranosyl*-*6-*O*-β-d-glucopyranosyl-β-d-glucopyranosyl) oxy]*ent*-kaur-16-en-19-oic acid (rebaudioside Z_1_) and **4** 13-[(2-*O*-β-d-glucopyranosyl*-*3-*O*-β-d-glucopyranosyl-β-d-glucopyranosyl) oxy]*ent*-hydroxyatis-16-en-19-oic acid. Three known compounds were purified in gram quantities and identified as rebaudiosides A (**5**), H (**6**) and J (**7**). Chemical structures were unambiguously elucidated using different approaches, namely HRESIMS, HRESI-MS/MS, and 1D-and 2D-NMR spectroscopic data. Additionally, a high-quality crystal of iso-stevioside was grown in methanol and its structure confirmed by X-ray diffraction.

## 1. Introduction

Diabetes is a chronic disease that affected 422 million of people worldwide in 2014 and is increasing considerably every year. Diabetes is characterized by insulin deficit or insulin insensitivity and consequently produces high blood sugar levels which are associated with other disorders [1,2]. Reducing or eliminating sugar consumption and replacing sucrose with healthier sweeteners is one approach to prevent and control diabetes.

*Stevia rebaudiana* (Bertoni) Bertoni and its steviol glycosides were conferred Generally Recognized as Safe status by the United States Food and Drug Administration in 2008 and in the European Union in 2011. Steviol glycosides from this Asteraceae plant have been gaining interest from companies and the general public since these natural compounds are calorie free sweeteners and have shown higher sweetener potency than sucrose [3,4]. Additionally, it was recently suggested that rebaudioside A potentiates the activity of a Ca^2+^ cation channel expressed in type II taste receptor cells and pancreatic β-cells (TRPM5) enhancing glucose-induced insulin secretion in a dependent manner. However, regardless of the potential of rebaudioside A as a sugar substitute to prevent and treat type 2 diabetes [5], the lingering aftertaste of this tetra-glucopyranosyl sugar substitute makes it unattractive to consumers.

In our continuing efforts to discover novel sugar substitutes with potential refined organoleptic properties, we describe herein the isolation and structure elucidation of two new tetra-glucopyranosyl diterpene glycosides, the preparation of their saponification products together with the isolation of three known compounds in gram quantities.

## 2. Results and Discussion

### Structure Elucidation

Two new compounds were isolated from a commercial extract of *Stevia rebaudiana* using reversed-phase and high performance normal-phase chromatography [6]. Recently, two approaches have been described for a rapid detection of novel oligosaccharide arrangements linked at position C-13 in steviol glycosides and infer their C-19 linkage. One is based on tandem mass spectrometry dissociation patterns with ranging collision energies [7]. Thus, steviol glycosides with one monosaccharide unit or a less hindered disaccharide linked at C-19 (e.g., rebaudiosides I and U with 1-3 and 1-6 linkages, respectively) cleave the C-19 ester linkage with low collision energies (10 eV) while more hindered disaccharides (1-2 linkages) cleave with higher collision energies (40 eV).

Compound **1** was purified as an amorphous off-white solid with [α]^25^_D_ −28.0 (*c* 0.1, MeOH). HR-ESIMS and HR-ESIMS/MS data of compound **1** showed a molecular ion at *m*/*z* 965.4208 [M − H]^−^ (calculated *m*/*z* 965.4235 [M − H]^−^), suggesting a molecular formula C_44_H_70_O_23_. Both, the deprotonated molecular ion and an intense product ion *m*/*z* = 803.3703 Da resulted from the loss of one hexose at C-19 ([M − H] − H_2_O − 162 Da)^−^ were observed at 10 eV. Further sequential loss of three hexoses ([M − H] − H_2_O – 3 × 162 Da)^−^ *m*/*z* = 641.3185, *m*/*z* = 479.2635 and *m*/*z* = 317.2146 from the C-13 moiety was observed at 70 eV. Acid hydrolysis of **1** furnished a mixture of aglycones and only D-glucose which was identified by comparison of the HPLC retention times of thiocarbamoyl thiazolidine derivatives prepared from sugar standards as previously described [8,9]. Hence compound **1** is a rebaudioside A isomer.

The second approach was based on comparing retention times of a pure steviol glycoside and its saponification product with those reported by RP-C18 HPLC [10]. Saponification condition was helpful to detect single monosaccharide, di and oligosaccharides linked at position C-19. Thus, compound **1** showed a retention time of 3.94 min that did not match with any previously reported steviol glycosides in a RP-C18 HPLC method [10]. The aglycone-C13 moiety was produced by mild alkaline hydrolysis corroborating the linkage of a single sugar at position C-19 (9.32 min) as deduced by MS. The aglycone with the C-13 portion showed an HPLC retention time that did not match with any of the steviol glycosides reported with a free carboxylic acid at C-19, suggesting the structural novelty of this compound, probably, in the C-13-oligosaccharide.

The assignments of the signals of the steviol aglycone started by identifying H-18 as the methyl protons coupling with C-19, which is the most deshielded carbon. H-18 also couples with a quaternary carbon, C-4, with methylene carbon, C-3, and with a methine carbon, C-5. The sequence H-3–H-2–H-1 could be followed in the COSY spectrum, and the protons on ring A assigned as axial or equatorial based on the number of large couplings. Both C-1 and C-5 couple with methyl protons at position 20, which also couples with a quaternary carbon, C-10 and with a methine carbon, C-9. The sequences H-6—H-7—H-9 and H-9—H-11—H-12 could be followed in the COSY spectrum. The C/D rings were supported by the HMBC correlations of H-9 with C-11, C-12, C-14 and C-15; H-14 with C-13, C-15 and C-16; and H-17 with C-13, C-15 and C-16.

We also observed four anomeric protons at δ 5.14, 5.19, 5.30, and 6.12 ppm supporting the MS information, all of them showing beta linkages.

**Figure 1:**
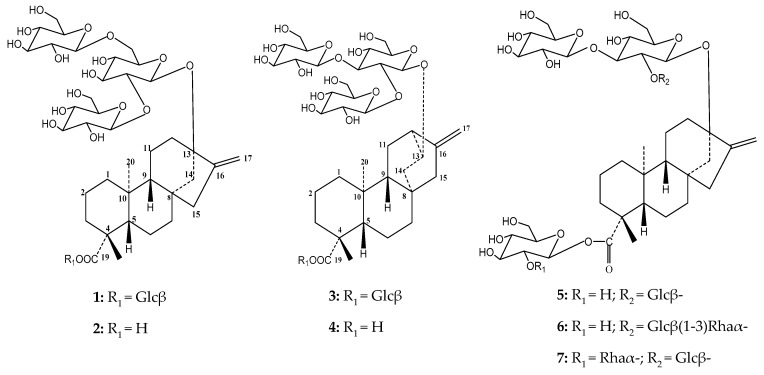
Chemical structures of purified compounds rebaudioside Z **(1)** and 13-[(2-*O*-β-d-glucopyranosyl*-*3-*O*-β-d-glucopyranosyl-β-d-glucopyranosyl)oxy]*ent*-hydroxyatis-16-en-19-oic acid-β-d-glucopyranosy ester (**3**) and chemically modified rebaudioside Z_1_ (**2**) and 13-[(2-*O*-β-d-glucopyranosyl*-*3-*O*-β-d-glucopyranosyl-β-d-glucopyranosyl) oxy]*ent*-hydroxyatis-16-en-19-oic acid (**4**), rebaudioside A (**5**), rebaudioside H (**6**) and rebaudioside J (**7**).

The positions of attachment of the sugar moieties were established based on the 2D-NMR HMBC spectrum. ^3^*J* HMBC correlations between anomeric proton H-1′ (δ 6.12 ppm) and C-19 (δ 177.7 ppm) confirmed the attachment of one glucose unit at C-19. The position of H-2′ was confirmed through the COSY correlation between H-2′ (δ 4.17 ppm) and H-1′ (δ 6.12 ppm). Additionally, the ^3^*J* HMBC correlations between H-1′′ (δ 5.19 ppm) and C-13 (δ 86.4 ppm) confirmed the attachment of the first glucose unit at C-13. The connections of other two glucoses were also established through ^3^*J* HMBC correlations between H-1′′′ (δ 5.30 ppm), H-1′′′′ (δ 5.14 ppm) with C-2′′ (δ 84.4 ppm) and C-6′′ (δ 70.3 ppm) respectively. In the same way, the position of H-2′′ was confirmed through the gDQCOSY correlation between H-2′′ (δ 4.23 ppm) and H-1′′ (δ 5.19 ppm). The positions of H-2 of the sugars linked at position C-13′ were confirmed through their COSY correlations between H-2′′ (δ 4.23 ppm) and H-1′′ (δ 5.19 ppm), H-2′′′ (δ 4.11 ppm) and H-1′′′ (δ 5.30 ppm) and H-2′′′′ (δ 4.06 ppm) and H-1′′′′ (δ 5.14 ppm). Compound **1** was named as 13-[(2-*O*-β-D-glucopyranosyl*-*6-*O*-β-D-glucopyranosyl-β-D-glucopyranosyl)oxy]*ent*-kaur-16-en-19-oic acid-β-D-glucopyranosy ester (rebaudioside Z) and assigned the structure shown in Figure 1. ^1^H- and ^13^C-NMR chemical shifts are shown in Table 1 and Table 2, respectively, and are typical signals of the *ent*-kaurene core [11].

Compound **2** was prepared through mild alkaline hydrolysis of **1** to afford a new rebaudioside B isomer with a Glcβ(1-6)[Glcβ(1-2)]Glcβ_1_- arrangement at C-13 with retention of 9.32 min in the RP-C18 HPLC method that did not match with any of the previously reported steviol-C13 oligosaccharides [10]. HRESIMS/MS experiment of compound **2** showed a molecular ion at *m*/*z* 803.3721 [M − H]^−^ (calculated *m*/*z* 803.3702 [M − H]^−^), suggesting a molecular formula C_38_H_60_O_18_. A sequential loss of three glucoses units at C-13 portion were observed at 70 eV collision energy: *m*/*z* 641.3196 (−162 Da), *m*/*z* 479.2648 (−162 Da) and *m*/*z* 317.2115 Da. ^1^H-NMR showed three anomeric protons corresponding to the oligosaccharide portion linked at position C-13 (δ_H_ 4.59; 4.61 and 4.62 ppm). The free carboxylic acid at position C-19 was evidenced by the signal at 184.1 ppm in the ^13^C-NMR, less shielded than the observed for compound **1**. Full structural assignment was performed using 1D- and 2D-NMR (DQCOSY, HSQC and HMBC) experiments. Compound **2** was named as 13-[(2-*O*-β-D-glucopyranosyl*-*6-*O*-β-D-glucopyranosyl-β-D-glucopyranosyl) oxy]*ent*-kaur-16-en-19-oic acid (rebaudioside Z_1_). ^1^H and ^13^C chemical shifts are presented in Table 1.

Compound **3** was purified as an amorphous off-white solid with [α]^25^_D_ −20.0 (*c* 0.1, MeOH). HR-ESIMS/MS data of compound **3** showed a molecular ion at *m*/*z* 965.4297 [M − H]^−^ (calculated *m*/*z* 965.4235 [M − H]^−^), suggesting a molecular formula C_44_H_70_O_23_. Both, the deprotonated molecular ion and an intense product ion *m*/*z* = 803.3735 Da resultant from the loss of one hexose at C-19 [M – H − H_2_O − 162 Da]^−^ were observed at 10 eV. Further sequential loss of three hexoses [M – H − H_2_O – 3 × 162 Da]^−^ *m*/*z* = 641.3195, *m*/*z* = 479.27.0 and *m*/*z* = 317.2141 from C-13 moiety was observed at 70 eV. Compound **3** showed a HPLC retention time of 4.35 min that did not match with any diterpene glycosides in the RP-C18 method previously reported [10]. It was hydrolyzed under mild alkaline conditions corroborating the presence of a single monosaccharide attached at position C-19. Retention time of the saponification product showed a peak at 10.5 min that did not match with any aglycone-C13 previously described [10]. The rapid elution of compound **3**, <7 min, suggested that it is a highly substituted glycoside with a single sugar unit linked at C-19. Acid hydrolysis of **3** furnished a mixture of aglycones and only D-glucose which was identified by comparison of the HPLC retention times of thiocarbamoyl thiazolidine derivatives prepared from sugar standards, as previously described [8,9].

**Figure 2:**
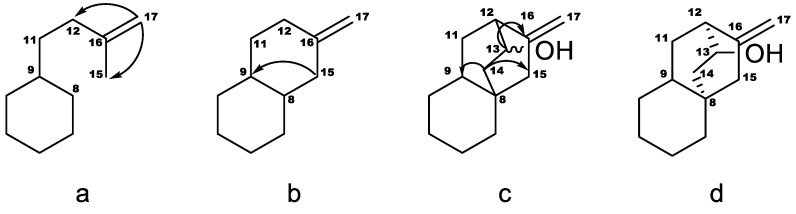
Structure elucidation for the 13(*S*)-hydroxyatisenoic acid. The arrows indicate cross-peaks in the HMBC spectrum between protons at the start position and carbons at the end position.

The aglycone signals were similar to those reported for steviol aglycone in compounds **1** and **2** except for δ_H_ 2.51 and δ_H_ 4.04 but signals were in good agreement with those reported for a similar aglycone previously found in *Stevia eupatoria* (Spreng.) Willd. and reported as 12-α-hydroxy-*ent*-kaur-16-en-19-oic acid based on the ^1^H- and ^13^C-NMR data together with chemical modifications [12]. Additionally, a triglucopyranosyl derivative isolated from *S. rebaudiana* (stevioside isomer) was also reported and was suggested a similar aglycone 12-α-hydroxy-*ent*-kaur-16-en-19-oic acid by comparison with previously reported data [13]. More recent NMR techniques allowed us to assign the aglycone as 13(*S*)-hydroxyastinoic acid. This type of aglycone was previously reported by converting microbiologically diterpene acids from *Helianthus* sp. with *Gibberella fujikuroi* [14]. The structure of the aglycone in **3** was inferred as described in Figure 2. The COSY spectrum reveals the sequence H-9–H-11ab–H-12. The coupling of H-17 with C-12 and C-15, seen in the HMBC spectrum, set fragment a. Coupling of H-15 with both C-8 and C-9 indicated that C-15 is bonded to C-8, as in fragment b. Couplings between the proton on the carbon carrying the oxygen (H-13, 4.04 ppm) with H-12 and H-14 were seen in the COSY spectrum, so they are vicinal, also corroborated for the HMBC correlation between H-13 and C-16. Protons in position 14 were identified by their coupling with H-13. They couple with C-8, C-9 and C-15, and therefore C-14 is bonded to C-8, as in fragment c. H-9 displayed a large coupling with C-14 and C-15, and the stereochemistry of fragment d was inferred. The stereochemistry of C-13 was assigned as *S* because of the large coupling of H-13 with C-16. The assignment of the diastereotopic protons was based on the large couplings between H-14 *pro-R* and C9, H-14 *pro-S* and C-15, H-15 *pro-S* and C-9, H-15 *pro-R* and C-14, H-11 *pro-S* and C-16, H-11 *pro-R* and C-13. A ROESY spectrum of compound **4**, in which the chemical shifts of the aglycone are very similar to those in **3**, confirmed this stereochemistry and these assignments, as it displayed nOes of H-20 with H-13 and H-14 *pro-R*, and of H-14 *pro-R* and H-11 *pro-R*.

Four anomeric protons at δ_H_ 4.56, 4.65, 4.81, and 5.43 ppm were also observed supporting the MS information, all of them showing beta linkages. ^1^H and ^13^C chemical shifts are shown in Table 1 and Table 2, respectively. The positions of attachment of the sugar moieties were established based on the HMBC spectrum. ^3^*J* HMBC correlations between anomeric proton H-1’ (δ 5.43 ppm) and C-19 (δ 176.8 ppm) confirmed the attachment of one glucose unit at C-19. The order of the protons in a sugar starting from the anomeric position was seen in the TOCSY spectra with increasing mixing times (Supporting Information). Additionally, the ^3^*J* HMBC correlations between H-1′′ (δ 4.56 ppm) and C-13 (δ 77.8 ppm) confirmed the attachment of the first glucose unit at C-13. The connections of the other two glucoses were also established through ^3^*J* HMBC correlations between H-1′′′ (δ 4.81 ppm), H-1′′′′ (δ 4.65 ppm) with C-2′′ (δ 79.0 ppm) and C-3′′ (δ 86.0 ppm) respectively. In the same way, position of H-2′′ was confirmed through the gDQCOSY correlation between H-2′′ (δ 3.64 ppm) and H-1′′ (δ 4.56 ppm). The order of the protons in a sugar starting from the anomeric position was seen in the TOCSY spectra with increasing mixing times, (Supporting Information). Compound **3** was named 13-[(2-*O*-β-D-glucopyranosyl*-*3-*O*-β-D-glucopyranosyl-β-D-glucopyranosyl)oxy]*ent*-hydroxyatis-16-en-19-oic acid-β-D-glucopyranosy ester and assigned structurally as shown in Figure 1.

Compound **4** was prepared through alkaline hydrolysis of **3** to afford a rebaudioside B isomer with a with a Glcβ(1-3)[Glcβ(1-2)]Glcβ_1_- arrangement at C-13 with retention of 10.5 min that did not match with any of the previously reported diterpene glycosides with a free carboxylic acid previously reported in the RP-C18 HPLC method [10]. HRESIMS/MS experiment of compound **4** showed a molecular ion at *m*/*z* 803.3682 [M − H]^−^ (calculated *m*/*z* 803.3702 [M − H]^−^), suggesting a molecular formula C_38_H_60_O_18_. A sequential loss of three glucoses units at C-13 portion at collision energy of 70 eV was observed: *m*/*z* 641.3162 (−162 Da), *m*/*z* 479.2703 (−162 Da) and *m*/*z* 317.2111 Da. ^1^H-NMR showed three anomeric protons corresponding to the oligosaccharide portion linked at position C-13 (δ_H_ 4.55; 4.65 and 4.81 ppm). The free carboxylic acid at position C-19 was evidenced by the signal at 184.4 ppm in the ^13^C-NMR, less shielded than that observed for compound **3**. Full structural assignment was performed using 1D- and 2D-NMR experiments. Compound **4** was named as 13-[(2-*O*-β-D-glucopyranosyl*-*3-*O*-β-D-glucopyranosyl-β-D-glucopyranosyl)oxy]*ent*-hydroxyatis-16-en-19-oic acid. ^1^H- and ^13^C-NMR chemical shifts are presented in Table 1. Additionally, glycosylation sites, sugar arrangements, retention times and [M − H]^−^ of compounds **1-5** including other related isomers were presented in Table 3. 

**Figure 4:**
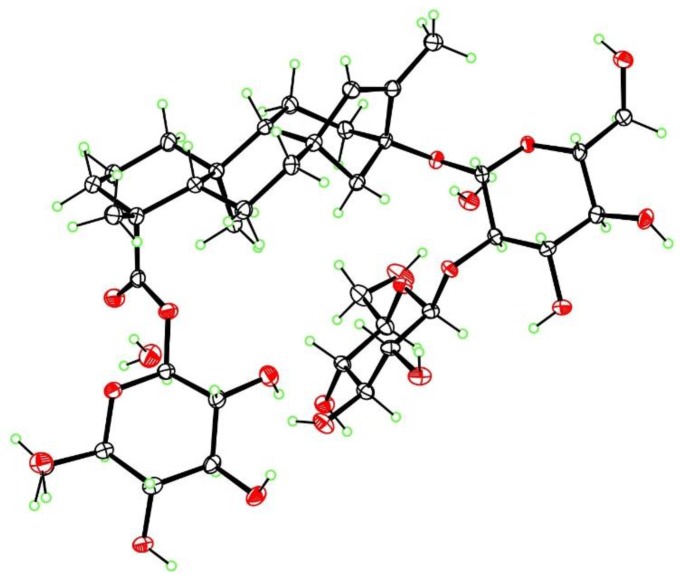
X-ray structure of iso-stevioside dihydrate, with ellipsoids at the 50% level (ORTEP).

Additionally, three known compounds were purified in gram quantities and identified as rebaudiosides A (**5**), H **(6**) and J (**7**). The purification of the degradation products formed under acidic condition from rebaudioside A and stevioside was recently reported [8]. Preparation of a high-quality crystal of iso-stevioside, one of the by-products, allowed the confirmation of its structure by X-ray diffraction (Figure 4)**.**

Rebaudioside A, one of the major compounds from *S. rebaudiana*, has found use as the main table-top and additive in beverages due to it being more than 200 times sweeter than sucrose and its potential human health benefits [5]. However, this compound certainly interacts with bitter taste receptors through several possible mechanisms by which the bitter aftertaste of rebaudioside A may suppress the sweet gustatory receptors activity. As far as we know, there is no complete study showing the relationship between structure-organoleptic properties of steviol glycosides. Only a couple of rebaudioside A isomers with sugar arrangement at position C-13 as follows Glcβ(1-6)[Glcβ(1-3)]-Glcβ_1_- and Glcβ(1-2)[Fruβ(1-3)]-Glcβ_1_- were reported [14]. The novel rebaudioside A isomers herein described, rebaudioside Z Glcβ(1-6)[Glcβ(1-2)]-Glcβ_1_- at C-13 and 13-[(2-*O*-β-D-glucopyranosyl*-*3-*O*-β-D-glucopyranosyl-β-D-glucopyranosyl) oxy]*ent*-hydroxyatis-16-en-19-oic acid-β-D-glucopyranosy ester Glcβ(1-3)[Glcβ(1-2)]-Glcβ_1_- at C-13 with a different aglycone may serve as models to provide important findings to better understand the relationship between sugar arrangement and positions with sweet/bitter flavors. To date, several steviol glycoside isomers have been isolated from *S. rebaudiana* mainly with different sugar arrangements, position of the attachment to the aglycone and also with different aglycones as is the case of stevioside (with Glcβ(1-2)Glcβ_1_- at C-13)/12-α-[(2-*O*-β-D-glucopyranosyl-β-D-glucopyranosyl) oxy]ent-kaur-16-en-19-oic acid-β-D-glucopyranosyl ester (Glcβ(1-2)Glcβ_1_- at C-12) [13]; rebaudioside E (with Glcβ(1-2)Glcβ_1_- at C-19) /rebaudioside Y (Glcβ(1-6)Glcβ_1_- at C-19) [15]; rebaudioside F (with Xylβ(1-2)[Glcβ(1-3)]-Glcβ_1_- at C-13)/rebaudioside R (Glcβ(1-2)[Glcβ(1-3)]-Xylβ_1_- at C-13)/rebaudioside F isomer (with Glcβ(1-2)[Xylβ(1-3)]-Glcβ_1_- at C-13) [14,16,17]; rebaudioside D (with Glcβ(1-2)Glcβ_1_- at C-19)/rebaudioside I (Glcβ(1-3)Glcβ_1_- at C-19) [17,18]. Additionally, several compounds differing in the type of sugar in a specific position could also be compared for better understanding of the organoleptic properties e.g., rebaudioside C (Rhaα(1-2)[Glcβ(1-3)]-Glcβ_1_- at C-13)/6-deoxyGlcβ(1-2)[Glcβ(1-3)]Glcβ_1_-/rebaudioside F [18,19]; rebaudioside A/Glcβ(1-2)[Fruβ(1-3)]-Glcβ_1_- at C-13 among others. Additionally, diterpene glycosides with an endocyclic double bond (C 15) could be compared with their exocyclic double bond isomers as in the case of iso-rebaudioside A/rebaudioside A; iso-stevioside/stevioside among other pairs of compounds.

## 3. Materials and Methods

### 3.1. Chemicals

Acetonitrile and water for HPLC and Silica gel 60 F254 HPTLC plates were purchased from EMD Millipore (Cincinnati, OH, USA). The bulk acetonitrile, methanol, methyl *tert*-butyl ether (MTBE) acetic acid, ethyl acetate, and isopropyl alcohol (IPA) were purchased from Reagents (Nashville, TN, USA). Flash silica was purchased from Sorbent Technologies (Atlanta, GA, USA).

### 3.2. General Experimental Procedures

The mass detector was a quadrupole time of flight (Model G6530A, Agilent, Palo Alto, CA, USA) equipped with an electrospray ionization interface and was controlled by Agilent software (A.05.00, Agilent MassHunter Work Station, Palo Alto, CA, USA). All acquisitions were performed under negative ionization mode with a capillary voltage of 3500 V. Nitrogen was used as nebulizer gas (30 psig) as well as drying gas at 10 L/min at drying gas temperature of 300 °C. The voltage of PMT, fragmentor and skimmer was set at 750 V, 100 V and 65 V respectively. Full scan mass spectra were acquired from *m*/*z* 100–1700. Data acquisition and processing was done using the MassHunter Workstation software (Qualitative Analysis Version B.07.00).

NMR spectra were acquired either at the University of Mississippi (Oxford, MS, USA) on an Avance NMR spectrometer (Bruker, Billerica, MA, USA) equipped with a Bruker 5 mm C13/H1-F19 cryoprobe or at the University of Florida (Gainesville, FL, USA) on an Inova spectrometer (Varian, Palo Alto, CA, USA) equipped with a Varian 5 mm H1/C13/P31-N15 indirect detection probe both operating at 500 MHz for proton and 125 MHz for carbon and using *z*-axis pulsed-field gradients. The temperature was set at 25 °C and chemical shifts (δ) were reported in ppm and referenced to tetramethylsilane or solvent signals using similar experimental conditions as previously reported [20].

### 3.3. Plant Material

The starting material was a partially processed commercially available extract of *Stevia rebaudiana* with Lot # SRE50-14091 purchased from American Mercantile (Memphis, TN, USA). HPLC comparison of that extract with several other *S. rebaudiana* extracts purchased from various sources showed high similarities, differing only in the relative concentrations of specific glycosides but not their presence or absence.

### 3.4. Isolation Procedure

Commercially available *S. rebaudiana* leaf extract (1.5 kg) was dissolved in methanol or 10% aqueous methanol at about 200 mg/mL and allowed to crystallize. The crystalline products were rebaudioside A and stevioside, which accounted for approximately 50% of the starting mass.

Pools rich in rebaudioside N from several large-scale chromatographies for isolation of rebaudioside C in quantity [21] were combined to obtain 140 g of extract. All this material was fractionated on a high efficiency reverse-phase chromatography column (7.5 i.d. ×50 cm, 10 µm spherical C18 gel) [20]. The column was loaded with the 140 grams dissolved in distilled water, the column eluted with 3 liters of 0.5% acetic acid in water and then switched to 15:85 acetonitrile: 0.1% acetic acid in water (3 liters); 25:75 acetonitrile: 0.1% acetic acid in water (3 liters); 40:60 acetonitrile: 0.1% acetic acid in water (3 liters); and column washed with 90:10 acetonitrile: 0.1% acetic acid in water and 100% methanol (1 liter each, washes combined and passed to waste.). 500 mL fractions collected and analyzed by HPLC. Similar fractions combined and fractions rich in rebaudioside N yielded ~22 grams. This pool was adsorbed onto celite (120 g), divided into three portions, each portion packed into a load column and chromatographed on a high efficiency normal-phase chromatography column (7.5 i.d. ×50 cm, 10 µm spherical silica gel) using Reb N mobile phase (Reb C mobile phase [100:18:14; EtOAc:MeOH:H_2_O with 0.1% AcOH] with additional 10 parts methanol and 10 parts water and 5 parts acetic acid). Column analysis [21] allowed combination of fractions rich in rebaudioside N. This pool spontaneously crystallized, the crystals filtered and the MLs dried, redissolved and a second crop obtained.

The supernatant second crop (7.02 g) was absorbed onto 70 g of Celite and subjected to a high efficiency normal-phase chromatography (7.5 i.d. ×50 cm, 10 µm spherical silica gel) with acetonitrile: H_2_O: AcOH (88:12:0.01 *v*/*v*/%). 2 × 1 L forerun were initially collected, followed by 48 fractions of 120 mL. All fractions were analyzed by HPLC methods and five main fractions were pooled based on results from column analysis [21]. *Chromatography* 1***,*** fraction 1.1 (0.093 g); fraction 2.1 (5.858 g); fraction 3.1 (0.64 g); fraction 4.1 (0.328 g) and fraction 5.1 (0.109 g). Fraction 2.1 was re-chromatographed in high efficiency normal-phase chromatography (7.5 i.d. ×50 cm) using MTBE: MeOH: H_2_O: AcOH (100:30:12.5:0.01). 2 × 1 L forerun and 40 × 120 mL fractions were collected and analyzed by HPLC. Five main fractions were selected by column analysis [21]. *Chromatography* 2*,* fraction 1.2 (1.3 g); fraction 2.2 (2.09 g); fraction 3.2 (1.49 g); fraction 4.2 (0.088 g) and fraction 5.2 (0.065 g). Fraction 1.2 (1.3 g) was chromatographed in a high efficiency reversed-phase column (7.5 i.d. ×50 cm, 10 µm spherical C18 gel) using ACN:H_2_O:AcOH (25:75:0.01). 2 × 1 L forerun and 25 × 120 mL fractions were collected and analyzed by HPLC. Column analysis [21] allowed us to select two main fractions. *Chromatography* 3***,*** fraction 1.3 (0.208 g); fraction 2.3 (0.922 g). Fraction 2.2 (2.09 g) and fraction 2.3 (0.922 g) were combined and digested with MeOH to afford 2.2 g of solids which were chromatographed in a high efficiency reversed-phase column (7.5 i.d. ×50 cm) using ACN:H_2_O:AcOH (23:77:0.01). 6 × 1 L + 1 × 0.7 L forerun, and 37 × 120 mL fractions were collected and analyzed by HPLC. Column analysis allowed us to select three main fractions. *Chromatography* 4***,*** fraction 1.4 (0.546 g); fraction 2.4 (0.713 g) and fraction 3.4 (1.165 g). Fraction 3.4 (1.165 g) was absorbed onto 10 g of celite and chromatographed in high efficiency normal-phase chromatography (7.5 i.d. ×50 cm) using “Reb C” mobile phase 2% MeOH [Reb C mobile phase = EtOAc:MeOH:H_2_O:AcOH (100:18:14:0.1; *v*/*v*/*v*/%)]. 1 × 1 L forerun and 48 × 120 mL fractions were collected and analyzed by HPLC. Column analysis allowed us to select five main pools. *Chromatography* 5*,* fraction 1.5 (67.1 mg); fraction 2.5 (135 mg); fraction 3.5 (226 mg); fraction 4.5 (179 mg) and fraction 5.5 (179 mg). Compound **1** (226 mg; 0.02% yield) was obtained from fraction 3.5.

Fractions rich in rebaudioside H were pooled (3.7 g) and chromatographed over a RP-C18 (7.5 i.d. ×50 cm) column with H_2_O:AcOH (100:0.1 *v*/*v*/%) and ACN:H_2_O:AcOH (5:95:0.1 *v*/*v*/%) to afford seven main fractions. *Chromatography* 6***,*** fraction 1.6 (175 mg); fraction 2.6 (120 mg); fraction 3.6 (108 mg); fraction 4.6 (169 mg); fraction 5.6 (1.8 g, rebaudioside H); fraction 6.6 (156 mg) and fraction 7.6 (13 mg). *Chromatography* 7, fraction 1.6 (175 mg) was submitted to a RP-C18 (250 × 10 mm, 5 µm) chromatography using H_2_O: AcOH (100:0.1) and ACN: H_2_O: AcOH (10:90:0.1) to afford two main fractions., fraction 1.7 (110 mg; 0.007% yield) and fraction 2.7 (48.3 mg). Compound **3** was obtained from fraction 1.7. Additionally, several hundred grams from the initial crystallization and subsequent chromatographies of rebaudioside A after processing 1.5 kg of commercial extract. Rebaudiosides J (1 g) and H (1.8 g) were also isolated.

### 3.5. Alkaline Hydrolysis of Compounds ***1*** and ***3***

Compounds **1** (40 mg) and **3** (30 mg) were heated individually with NaOH (1 N) at 80 °C over 1 h. Each reaction mixture was cooled over 5 min and neutralized with two or three drops of acetic acid glacial [18] with further cleanup through a Strata RP-C18-E cartridge (500 mg/6 mL) (Phenomenex, Torrance, CA, USA). Elution with a stepwise gradient of 1.5 mL volume each, water, acetonitrile: water (2:8) and methanol to produce clean compounds **2** (27 mg; 68% yield) and **4** (15 mg; 50% yield).

### 3.6. Physicochemical Parameters of Compounds

*Rebaudioside Z* (**1**): Amorphous off-white solid; [α]^25^_D_ −28.0 (*c* 0.1, MeOH). HR-ESIMS/MS *m*/*z* 965.4222 [M − H]^−^ (calculated for C_44_H_70_O_23_, 966.4309), *m*/*z* 803.3703 at 10 eV collision energy, loss of one hexose (−162 Da) from C-19 moiety, *m*/*z* 641.3185 (−162 Da), 479.2635 (−162 Da) and 317.2146 (−162 Da), loss of three hexoses from C-13 moiety at 70 eV collision energy. ^1^H- and ^13^C-NMR spectroscopic data are shown in Table 1 and Table 2.

*Rebaudioside Z_1_* (**2**): Amorphous off-white solid; [α]^25^_D_ −46.0 (*c* 0.1, MeOH). HR-ESIMS/MS *m*/*z* 803.3721 [M − H]^−^ (calculated for C_38_H_60_O_18_, 804.3781), *m*/*z* 641.3196 (−162 Da), 479.2648 (−162 Da) and 317.2115 (−162 Da), loss of three hexoses from C-13 moiety at 70 eV collision energy. ^1^H- and ^13^C-NMR spectroscopic data are shown in Table 1 and Table 2.

*13-[(2-O-β-D-Glucopyranosyl-3-O-β-D-glucopyranosyl-β-D-glucopyranosyl) oxy]ent-hydroxyatis-16-en-19-oic acid-β-d-glucopyranosy ester* (**3**): Amorphous off-white solid; [α]^25^_D_ −22.0 (c 0.1, MeOH), HR-ESIMS/MS *m*/*z* 965.4297 [M − H]^−^ (calculated for C_44_H_70_O_23_, 966.4309), *m*/*z* 803.3735 at 10 eV collision energy (−162 Da), loss of one hexoses from C-19 moiety, *m*/*z* 641.3195 (−162 Da), 479.2703 (−162 Da) and 317.2141 (−162 Da), loss of three hexoses from C-13 moiety at 70 eV collision energy. ^1^H- and ^13^C-NMR spectroscopic data are shown in Table 1 and Table 2.

*13-[(2-O-β-D-Glucopyranosyl-3-O-β-D-glucopyranosyl-β-D-glucopyranosyl) oxy]ent-hydroxyatis-16-en-19-oic acid* (**4**): Amorphous off-white solid; [α]^25^_D_ −20.0 (*c* 0.1, MeOH). HRESIMS/MS *m*/*z* 803.3682 [M − H]^−^ (calculated for C_38_H_60_O_18_, 803.3702), *m*/*z* 641.3162 (−162 Da), *m*/*z* 479.2703 (−162 Da) and *m*/*z* 317.2111 Da at 70 eV collision energy. ^1^H- and ^13^C-NMR spectroscopic data are shown in Table 1 and Table 2.

### 3.7. RP-C18 HPLC Analysis

Analyses were performed with a Hewlett Packard Agilent 1100 Series system equipped with a G1311A quaternary pump, a G1322 degasser, a G1316A oven, G1313A autosampler and a G1315A diode array detector. Acetonitrile and water for HPLC were purchased from EMD Millipore (Cincinnati, OH, USA). The elution was performed with 0.01 M phosphoric acid (A) and acetonitrile (B) with a flow rate set at 1 mL/min. All the analyses were performed with Phenomenex columns. After each analysis, the column was washed and equilibrated appropriately. 10 µL of compound **1**–**4** were injected in the RP C-18 Luna (2), Phenomenex (250 × 4.6 mm, 5 µm) column at 30 °C. The elution was performed using gradient of elution as follows: 0–5 min, 32% B; 5–13 min, 32–41% B; 13–16 min, 41–43% B; 16–17 min: 43–50% B; 17–23 min, 50% B. The chromatogram was recorded at 205 nm and the flow rate set at 1 mL/min [10].

### 3.8. Determination of the Sugar Unit Absolute Configuration

Compounds **1** and **3** (1 mg) were hydrolyzed with HCl (1 N) at 80 °C over 2 h followed by liquid-liquid partition with ethyl acetate (2 × 1 mL). The aqueous layer was neutralized with silver carbonate and the supernatant was recovered and heated with l-cysteine methyl ester in pyridine for 1 h at 60–70 °C. The mixture was dried in a vacuum oven at 40 °C. After dryness, 400 µL of pyridine and 100 µL of phenylisothiocyanate were added and heated for an additional hour at 60–70 °C to form the thiocarbamoyl thiazoline derivatives. Reaction mixtures were analyzed by the HPLC method previously reported [9]. The absolute configuration of the sugars was determined by comparison of the HPLC retention times of the prepared thiocarbamoyl thiazolidine derivatives with appropriate standards.

### 3.9. X-ray Crystallography of Iso-Stevioside

The crystal structure and absolute configuration of iso-stevioside dihydrate were determined from a colorless crystal of dimensions 0.45 × 0.14 × 0.02 mm, using data collected at T = 90 K with Cu Kα radiation on an APEX-II DUO CCD diffractometer (Bruker, Madison, WI, USA) equipped with a microfocus source and a Cryostream cooler (Oxford, Cryosystems, Oxford, UK). The structure was solved using the program SHELXS-97 (University of Göttingen, Germany) and refined anisotropically by full-matrix least-squares on F^2^ using SHELXL-2014/7 (University of Göttingen, Germany) [22]. All H atoms were visible in difference maps, but were placed in idealized positions for the refinement, except for those of OH groups and water molecules, which were refined. The absolute configuration was determined from the Flack [23] parameter of 0.01(5) based on resonant scattering of the light atoms and 2804 quotients. The reported configuration has C4(*R*), C5(*S*), C8(*R*), C9(*R*), C10(*S*), C13(*S*) and is in agreement with the known configurations of the β-D-glucopyranose moieties. Crystal data: C_38_H_60_O_18_·2H_2_O, Mr = 840.89, monoclinic space group P2_1_, a = 13.2330(6) Å, b = 8.1081(4) Å, c = 19.1556(8) Å, β = 105.655(2)°, V = 1979.05(16) Å^3^, Z = 2, Dx = 1.411 g cm^−3^, θ_max_ = 68.3°, R = 0.031 for all 6901 unique data and 571 refined parameters. Supplementary crystallographic data for iso-stevioside dihydrate are contained in Cambridge Structural Database deposition CCDC-1879103; this data can be obtained free of charge via www.ccdc.cam.ac.uk/conts/retrieving.html (or from the Cambridge Crystallographic Data Centre, 12 Union Road, Cambridge CB2 1EZ, UK; fax: (+44) 1223-336-033 or e-mail: deposit@ccdc.cam.ac.uk)

## 4. Conclusions

Two new rebaudioside A isomers, rebaudiosides Z (**1**) and 13-[(2-*O*-β-D-glucopyranosyl*-*3-*O*-β-D-glucopyranosyl-β-D-glucopyranosyl) oxy]*ent*-hydroxyatis-16-en-19-oic acid-β-D-glucopyranosy ester (**3**) were isolated from a partially processed commercial extract of *S. rebaudiana* and two new rebaudioside B isomers, rebaudiosides Z_1_ and 13-[(2-*O*-β-D-glucopyranosyl*-*3-*O*-β-D-glucopyranosyl-β-D-glucopyranosyl) oxy]*ent*-hydroxyatis-16-en-19-oic acid were prepared and purified for the first time, respectively. An additional three known compounds, rebaudiosides A, H and J, were purified in gram quantities. Scarce rebaudioside A isomers have been reported from *Stevia rebaudiana* differing in sugar arrangement and type of sugar at position C-13 (Glcβ(1-6)[Glcβ(1-3)]-Glcβ_1_-) and Glcβ(1-2)[Fruβ(1-3)]-Glcβ_1_-. However, herein we describe the occurrence of two rebaudioside A isomers with (Glcβ(1-6)[Glcβ(1-2)]-Glcβ_1_-) and (Glcβ(1-3)[Glcβ(1-2)]-Glcβ_1_-) at position C-13. However, compound **3** showed a different aglycone and was found to be a 13(*S*)-hydroxyatisenoic acid type. This finding may contribute to better understanding of the relationship between sweet/bitter taste of Stevia glycosides with their sugar arrangements. Several new compounds have been reported in recent years, however, a systematic study comparing organoleptic properties with structure of diterpene glycosides is still not available in literature.

## Figures and Tables

**Figure 3:**
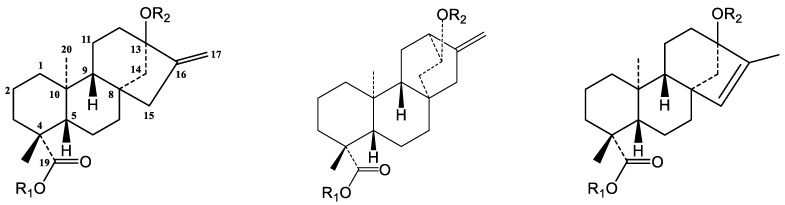
Chemical structures of steviol (I), hydroxyatisenoic acid (II) and *endo*-steviol aglycone (III).

**Table 1:** ^1^H chemical shifts of diterpene glycosides **1**–**4**.

Moiety	Position	1 ^a^	2 ^b^	3 ^b^	4 ^b^
		δ (ppm)	δ (ppm)	δ (ppm) ^c^	δ (ppm) ^c^
Aglycone	1	0.73, 1.73	0.95, 2.13	0.92, 1.59	0.85, 1.56
	2	2.20, 1.43	1.96, 1.37	1.92, 1.40	2.01, 1.30
	3	2.28, 1.79	1.97, 1.54	2.20, 1.09	2.14, 0.88
	5	1.03	1.01	1.12	0.90
	6	1.91, 2.50	1.87, 1.87	1.78, 2.05	1.80, 1.98
	7	1.28, 1.28	1.52; 1.40	1.57, 1.16	1.53, 1.13
	9	0.87	0.96	1.09	1.04
	11	1.68, 1.68	1.79,1.63	1.63, 1.45	1.60, 1.44
	12	1.02, 2.35	0.83, 1.86	2.51	2.49
	13	-	-	4.04	4.05
	14	1.97, 2.74	1.51, 2.27	1.15, 2.51	1.14, 2.51
	15	2.06, 2.06	2.05, 2.10	1.89, 2.11	1.86, 2.08
	17	5.10, 5.73	4.85, 5.24	4.70, 4.84	4.68,4.82
	18	1.23	1.14	1.24	1.11
	20	1.31	1.03	0.89	0.95
Glcβ-C_19_	1′	6.12	-	5.43	-
	2′	4.17	-	3.39	-
	3′	3.98	-	3.43	-
	4′	4.33	-	3.39	-
	5′	4.22	-	3.39	-
	6′	4.43, 4.57	-	3.85, 3.71	-
Glc-C_13_	1′′	5.19	4.59	4.56	4.55
	2′′	4.23	3.60	3.65	3.64
	3′′	4.27	3.54	3.71	3.71
	4′′	4.45	3.35	3.38	3.42
	5′′	4.08	3.17	3.34	3.30
	6′′	4.51, 4.78	3.78, 4.10	3.91, 3.68	3.85, 3.70
Glc(1–2)	1′′′	5.30	4.62	4.81	4.81
	2′′′	4.11	3.30	3.19	3.18
	3′′′	4.23	3.46	3.35	3.34
	4′′′	4.28	3.35	3.22	3.21
	5′′′	3.91	3.36	3.33	3.30
	6′′′	4.22, 4.57	3.68, 3.82	3.87, 3.66	3.85, 3.65
Glcβ(1-X) ^d^	1′′′′	5.14	4.61	4.65	4.65
	2′′′′	4.06	3.21	3.28	3.27
	3′′′′	4.27	3.46	3.39	3.38
	4′′′′	4.12	3.35	3.31	3.33
	5′′′′	4.03	3.36	3.37	3.36
	6′′′′	4.22, 4.57	3.68, 3.82	3.90, 3.66	3.89, 3.65

(**1**) rebaudioside Z; (**2**) rebaudioside Z_1_; (**3**) 13-[(2-*O*-β-d-glucopyranosyl*-*3-*O*-β-d-glucopyranosyl-β-d-glucopyranosyl) oxy]*ent*-hydroxyatis-16-en-19-oic acid-β-d-glucopyranosy ester; (**4**) 13-[(2-*O*-β-d-glucopyranosyl*-*3-*O*-β-d-glucopyranosyl-β-d-glucopyranosyl)oxy]*ent*-hydroxyatis-16-en-19-oic acid. All the sugars showed β linkage evidenced for the coupling constants of the anomeric protons (7.6 and 8 Hz), ^a^ in Pyr-*d*_5_, ^b^ in MeOH-*d*_4_, ^c^ diastereotopic protons in the aglycone are given in the order *pro-R*, *pro-S*. Protons in position 6 of glucose were not assigned sterically. Protons in position 17 are given in the order *pro-E*, *pro-Z.* ^d^ X = 6 for compound **1** and **2**; X = 3 for compound **3** and **4**.

**Table 2:** ^13^C chemical shifts of diterpene glycosides **1**–**4**.

Moiety	Position	1 ^a^	2 ^b^	3 ^b^	4 ^b^
		δ (ppm)	δ (ppm)	δ (ppm)	δ (ppm)
Aglycone	1	41.2	40.1	39.5	40.5
	2	19.9	20.9	18.4	19.3
	3	36.8	39.0	37.7	39.4
	4	44.5	45.6	43.7	44.7
	5	57.8	58.7	57.1	57.6
	6	22.7	23.7	19.9	20.6
	7	42.2	43.1	38.8	39.4
	8	43.3	43.1	33.9	33.8
	9	54.3	55.3	51.0	51.2
	10	40.3	40.9	38.1	38.0
	11	21.1	21.5	26.3	26.3
	12	38.9	42.6	41.0	41.0
	13	86.4	88.3	77.8	77.8
	14	45.1	45.8	37.7	37.7
	15	48.0	48.8	47.4	47.7
	16	155.0	154.2	146.7	147.0
	17	105.4	105.7	108.0	107.8
	18	28.8	30.1	27.5	29.1
	19	177.7	184.1	176.8	184.4
	20	16.0	17.3	11.9	12.2
Glcβ-C_19_	1′	96.3	-	94.2	-
	2′	74.4	-	72.6	-
	3′	79.5	-	77.3	-
	4′	71.4	-	69.7	-
	5′	79.9	-	77.3	-
	6′	62.6	-	61.0	-
Glcβ-C_13_	1′′	98.3	97.7	100.5	100.3
	2′′	84.4	81.8	79.0	78.8
	3′′	78.6	78.5	86.0	85.9
	4′′	71.8	71.8	68.8	68.6
	5′′	77.8	77.8	76.1	76.1
	6′′	70.3	70.0	61.4	61.2
Glcβ(1-2)	1′′′	106.8	104.8	102.2	102.1
	2′′′	75.7	76.3	74.6	74.5
	3′′′	78.8	77.8	76.5	76.5
	4′′′	72.1	71.4	70.6	70.6
	5′′′	78.3	77.9	76.6	76.6
	6′′′	63.3	62.8	61.9	61.8
Glcβ(1-X)	1′′′′	105.9	104.6	103.1	103.1
	2′′′′	77.2	75.3	73.9	73.9
	3′′′′	78.9	77.9	76.8	76.8
	4′′′′	72.6	71.7	70.1	70.1
	5′′′′	78.5	78.3	76.8	76.8
	6′′′′	63.3	62.8	61.2	61.1

**(1)** Rebaudioside Z; **(2)** rebaudioside Z_1_; **(3)** 13-[(2-*O*-β-d-glucopyranosyl*-*3-*O*-β-d-glucopyranosyl-β-d-glucopyranosyl) oxy]*ent*-hydroxyatis-16-en-19-oic acid-β-d-glucopyranosy ester; **(4)** 13-[(2-*O*-β-d-glucopyranosyl*-*3-*O*-β-d-glucopyranosyl-β-d-glucopyranosyl) oxy]*ent*-hydroxyatis-16-en-19-oic acid. NMR spectra recorded in ^a^ Pyr-*d*_5_ and ^b^ MeOH-*d*_4._ X = 6 for compound **1** and **2**; X = 3 for compound **3** and **4**.

**Table 3:** Glycosylation sites, sugar arrangements, HPLC retention times, molecular weights and type of aglycones of rebaudioside A and B isomers.

DG	Glycosylation Sites			RT (min) ^a^		[M − H]^−^ (*m/z*)
	C-12 (R_2_)	C-13 (R_2_)	C-19 (R_1_)	DG	Agly ^b^	Experimental
Rebaudioside Z **(1)**	-CH_2_	Glcβ(1-2)[Glcβ(1-6)]-Glcβ_1_-	Glcβ_1_-	3.94	I	965.4222
Compound **3**	-CH	Glcβ(1-2)[Glcβ(1-3)]-Glcβ_1_- and -H	Glcβ_1_-	4.35	II	965.4297
Rebaudioside A	-CH_2_	Glcβ(1-2)[Glcβ(1-3)]-Glcβ_1_-	Glcβ_1_-	7.11	I	965.4178
Iso-rebaudioside A	-CH_2_	Glcβ(1-2)[Glcβ(1-3)]-Glcβ_1_-	Glcβ_1_-	7.91	III	965.4204
Rebaudioside Z_1_ **(2)**	-CH_2_	Glcβ(1-2)[Glcβ(1-6)]-Glcβ_1_-	H	9.32	I	803.3721
Compound **4**	-CH	Glcβ(1-2)[Glcβ(1-3)]-Glcβ_1_- and -H	H	10.5	II	803.3682
Rebaudioside B	-CH_2_	Glcβ(1-2)[Glcβ(1-6)]-Glcβ_1_-	H	14.32	I	803.3692
Iso-rebaudioside B	-CH_2_	Glcβ(1-2)[Glcβ(1-3)]-Glcβ_1_-	H	14.44	III	803.3682

^a^ Reversed-phase C18 high performance liquid chromatography method. ^b^ Aglycone structures are presented in Figure 3. Compound **3**: 13-[(2-*O*-β-d-glucopyranosyl*-*3-*O*-β-d-glucopyranosyl-β-d-glucopyranosyl) oxy]*ent*-hydroxyatis-16-en-19-oic acid-β-d-glucopyranosy ester; compound **4**: 13-[(2-*O*-β-d-glucopyranosyl*-*3-*O*-β-d-glucopyranosyl-β-d-glucopyranosyl) oxy]*ent*-hydroxyatis-16-en-19-oic acid.

## Supplementary Materials

The supporting information are available online.
