# Supplementary material for: Tetra-glucopyranosyl Diterpene ent-Kaur-16-en-19-oic Acid and ent-13(S)-Hydroxyatisenoic Acid Derivatives from a Commercial Extract of Stevia rebaudiana (Bertoni) Bertoni
Source: Molecules. 2018 Dec 15;23(12):3328. doi: 10.3390/molecules23123328 (PMC6321316; doi:10.3390/molecules23123328)
Supplement: Supplementary file 1 [file molecules-23-03328-s001.pdf]

SUPPLEMENTARY INFORMATION

Tetra-glucopyranosyl diterpene *ent*-kaur-16-en-19-oic acid and ent-13(*S*)-hydroxyatisenoic acid derivatives from a commercial extract of *Stevia rebaudiana* (Bertoni) Bertoni

Wilmer H. Perera,[1,2] Ion Ghiviriga,[3] Douglas L. Rodenburg,[1] Kamilla Alves,[1] Frank T. Wiggers,[4] Charles D. Hufford,[4,†] Frank R. Fronczek,[5] Mohamed A. Ibrahim,[4,6] Ilias Muhammad,[4] Bharathi Avula,[4] Ikhlas A. Khan[4] and James D. McChesney [1*]

1 Ironstone Separations, Inc., Etta, Oxford, Mississippi 38627, USA; wilmer.perera@gmail.com (W.H.P.); douglasrodenburg@yahoo.com (D.L.R.); Kamilla_07@yahoo.com (K.A.); jdmcchesney@yahoo.com (J.D.M.)

2 ORISE Fellow-Agricultural Research Service, U.S. Department of Agriculture, Natural Product Utilization Research Unit, University of Mississippi, Mississippi 38677, USA

3 Department of Chemistry, Gainesville, University of Florida, Florida 32611, USA

4 National Center for Natural Products Research, University of Mississippi, Mississippi 38677, USA, fwiggers@olemiss.edu (F.T.W.); mmibrahi@olemiss.edu(M.A.I.); milias@olemiss.edu (I.M.)

5 Department of Chemistry, Louisiana State University, Baton Rouge, Louisiana 70803, USA, ffroncz@lsu.edu

6 Chemistry of Natural Compounds Department, Pharmaceutical and Drug Industries Division, National Research Centre, Dokki, Giza 12622, Egypt

* Correspondence: jddmcchesney@yahoo.com; Tel.: (303)808-4104

† Charles D. Hufford deceased on May 15th, 2017.

Figure	Page
**Figure 1**. RP-C18 HPLC chromatogram, chemical structure and MS/MS spectrum of compound **1**	1
**Figure 2**. ^{1}H NMR spectrum of compound **1**	2
**Figure 3**. ^{13} C NMR spectrum of compound **1**	2
**Figure 4**. DEPT 135 spectrum of compound **1**	3
**Figure 5**. DQCOSY spectrum of compound **1**	3
**Figure 6**. HSQC spectrum of compound **1**	4
**Figure 7**. HMBC spectrum of compound **1**	4
**Figure 8**. TOCSY spectrum of compound **1**	5
**Figure 9**. RP-C18 HPLC chromatogram, chemical structure and MS/MS spectrum of compound **2**	6
**Figure 10**. ^{1}H NMR spectrum of compound **2**	7
**Figure 11**. ^{13} C NMR spectrum of compound **2**	7
**Figure 12**. DEPT 135 spectrum of compound **2**	8
**Figure 13**. DQCOSY spectrum of compound **2**	8
**Figure 14**. HSQC spectrum of compound **2**	9
**Figure 15**. HMBC spectrum of compound **2**	9
**Figure 16**. RP-C18 HPLC chromatogram, chemical structure and MS/MS spectrum of compound **3**	10
**Figure 17**. ^{1}H NMR spectrum of compound **3**.	11
**Figure 18**. DQCOSY spectrum of compound 3.	11
**Figure 19**. HSQC spectrum of compound 3 from 60 - 110 ppm.	12
**Figure 20**. HSQC spectrum of compound 3 from 10 – 60 ppm.	12
**Figure 21**. HSQC spectrum of compound 3 from 60 – 88 ppm.	13
**Figure 22**. HSQC spectrum of compound 3 from 60 – 63 ppm.	13

Figure	Page
**Figure 23**. HMBC spectrum of compound 3.	14
**Figure 24**. HMBC spectrum of compound 3 from 5 – 105 ppm.	14
**Figure 25**. HMBC spectrum of compound 3 from 10 – 80 ppm.	15
**Figure 26**. HMBC spectrum of compound 3 from 60 – 105 ppm.	15
**Figure 27**. ^{1}H-^{1}H band-selective TOCSY spectrum of compound 3 with a mixing time of 50 ms.	16
**Figure 28**. ^{1}H-^{1}H band-selective TOCSY spectrum of compound 3 with a mixing time of 70 ms.	16
**Figure 29**. ^{1}H-^{1}H band-selective TOCSY spectrum of compound 3 with a mixing time of 100 ms.	17
**Figure 30**. ^{1}H-^{1}H band-selective TOCSY spectrum of compound 3 with a mixing time of 150 ms.	17
**Figure 31**. RP-C18 HPLC chromatogram**,** chemical structure and MS/MS spectrum of compound **4**.	18
**Figure 32**. ^{1}H NMR spectrum of compound **4**.	19
**Figure 33**. DQCOSY spectrum of compound **4**.	19
**Figure 34**. DQCOSY spectrum of compound **4**.	20
**Figure 35**. HSQC spectrum of compound 4 from 0 - 120 ppm.	20
**Figure 36**. HSQC spectrum of compound 4 from 60 - 86 ppm.	21
**Figure 37**. HSQC spectrum of compound 4 from 10 - 60 ppm.	21
**Figure 38**. HMBC spectrum of compound 4 from 10 - 200 ppm.	22
**Figure 39**. HMBC spectrum of compound 4 from 10 - 80 ppm.	22
**Figure 40**: ^{1}H spectrum, bottom, and TOCSY 1D spectra with selective excitation at 4.55 ppm, and mixing times from lower to upper, 30, 60, 90, 120 and 150 ms.	23
**Figure 41**: ^{1}H spectrum, bottom, and TOCSY 1D spectra with selective excitation at 4.65 ppm, and mixing times from lower to upper, 30, 60, 90, 120 and 150 ms.	23
**Figure 42**: ^{1}H spectrum, bottom, and TOCSY 1D spectra with selective excitation at 4.81 ppm, and mixing times from lower to upper, 30, 60, 90, 120 and 150 ms.	24

Rebaudioside Z
(compound 1)

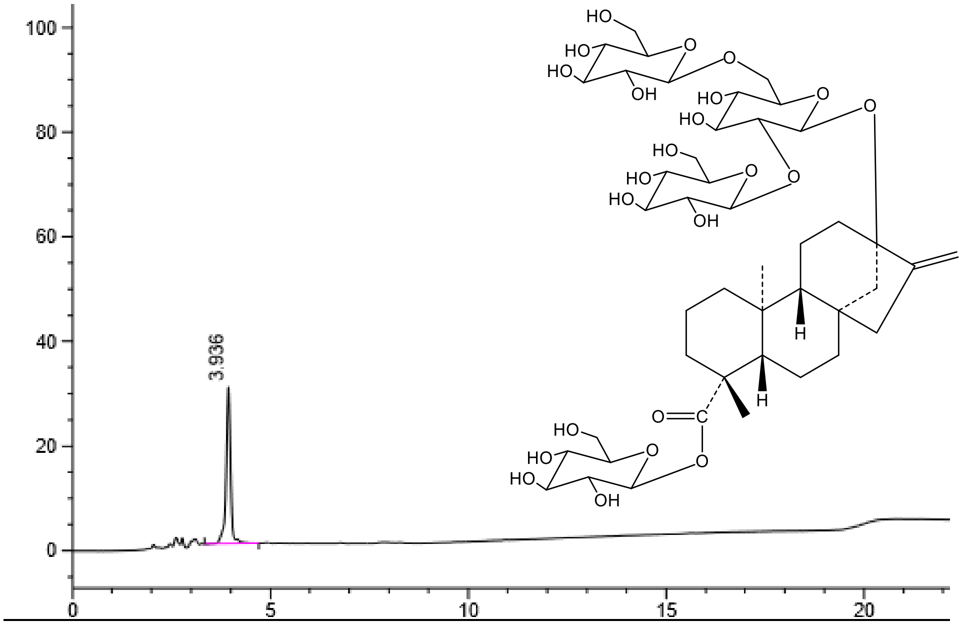

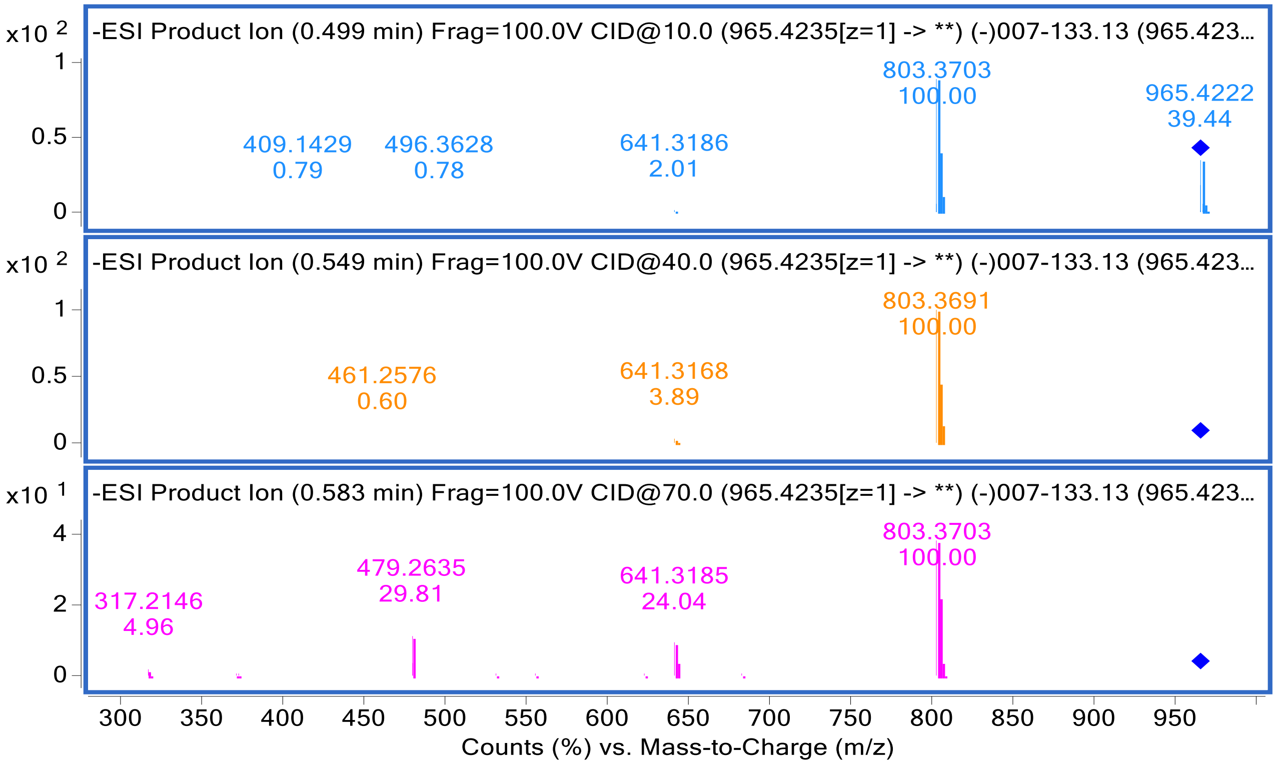

Figure 1. RP-C18 HPLC chromatogram**,** chemical structure and MS/MS spectrum of compound **1**.

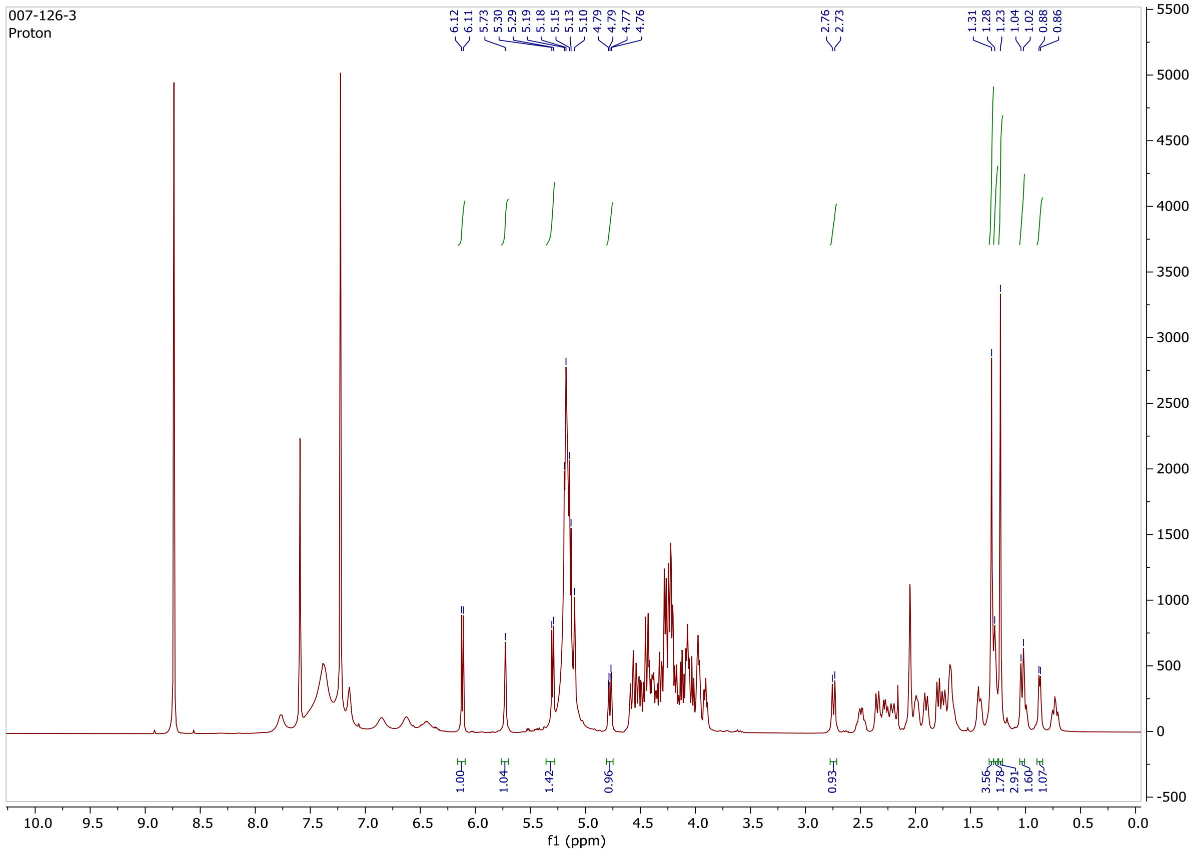

Figure 2. ^{1}H NMR spectrum of compound **1**.

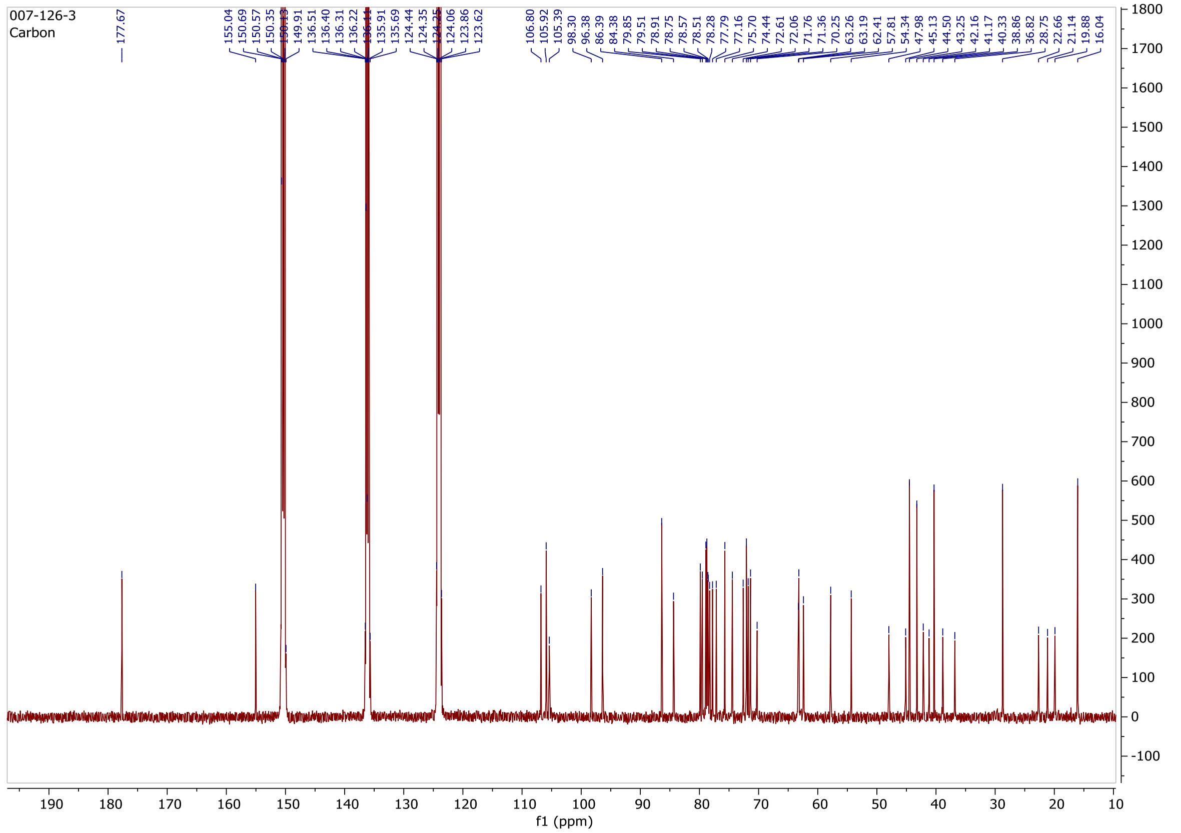

Figure 3. ^{13} C NMR spectrum of compound **1**.

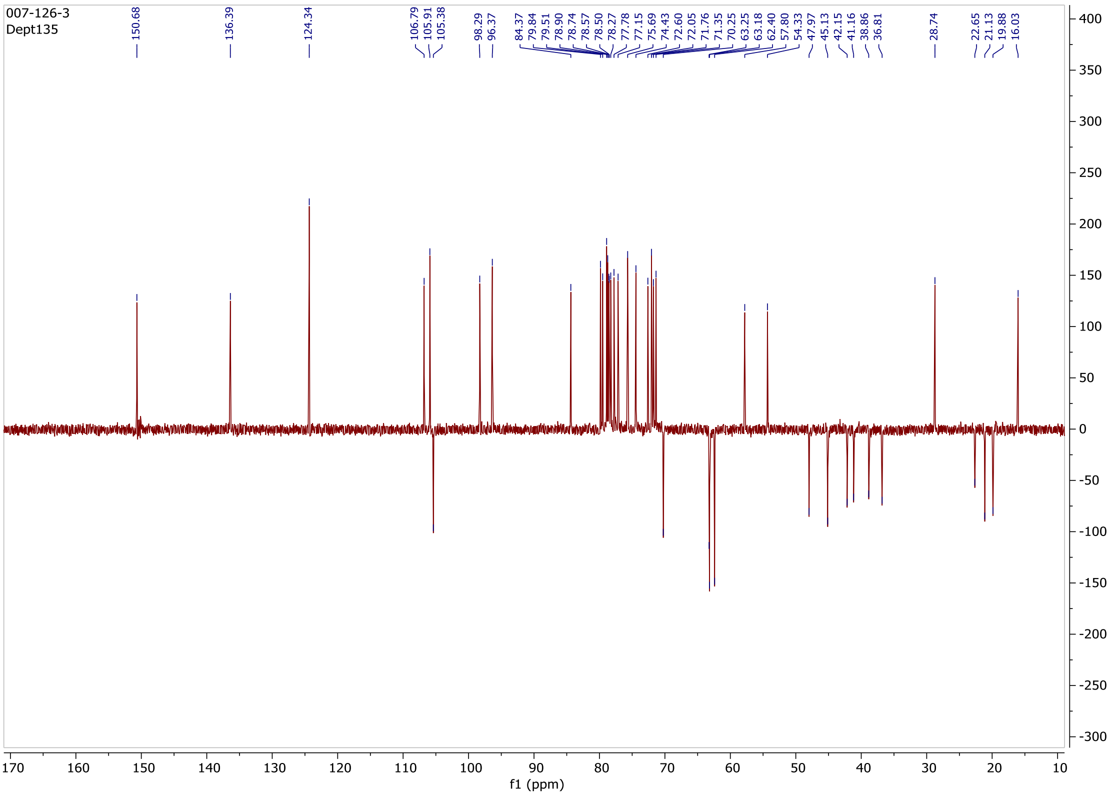

Figure 4. DEPT 135 spectrum of compound **1**.

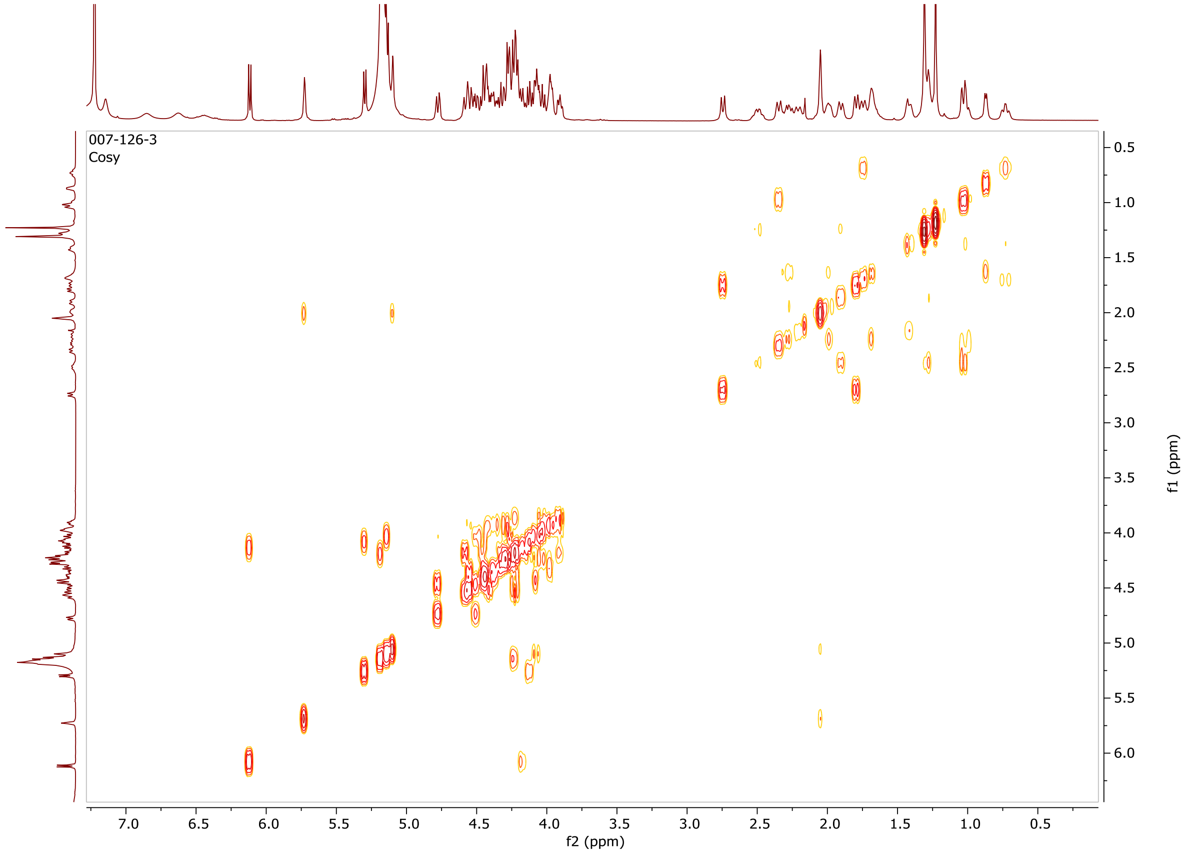

Figure 5. DQCOSY spectrum of compound **1**.

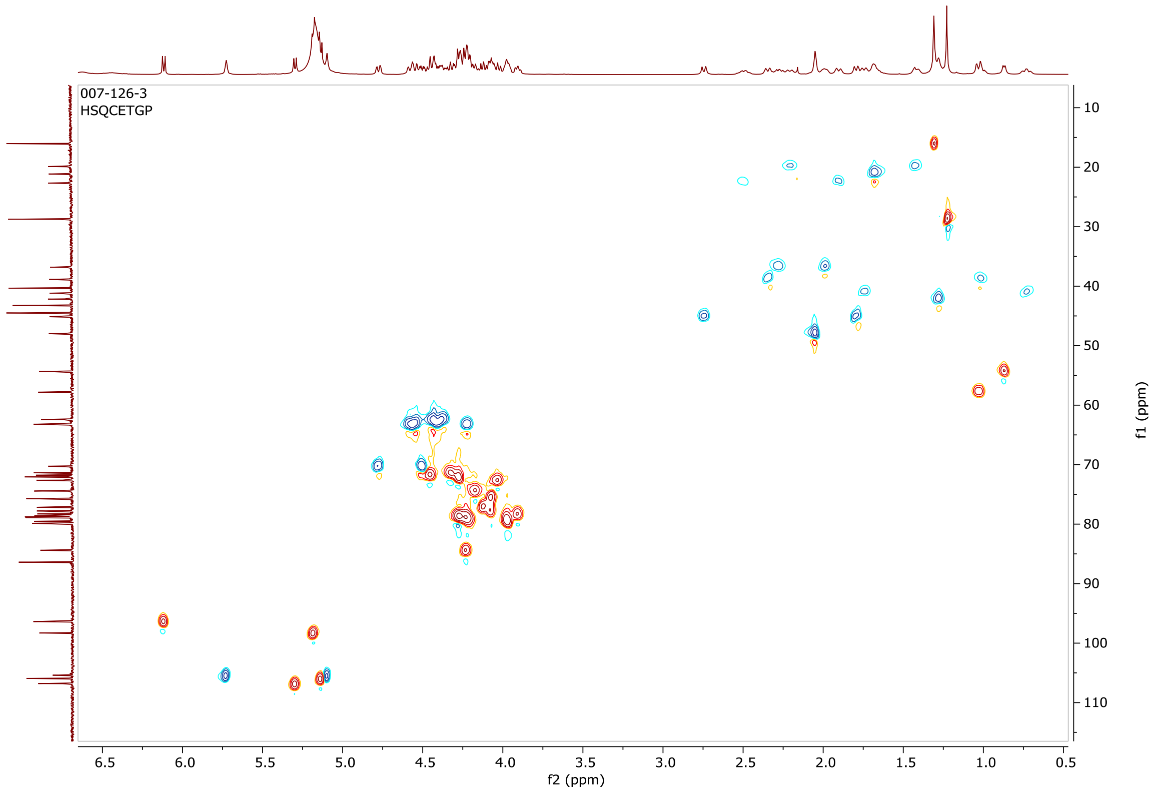

Figure 6. HSQC spectrum of compound **1**.

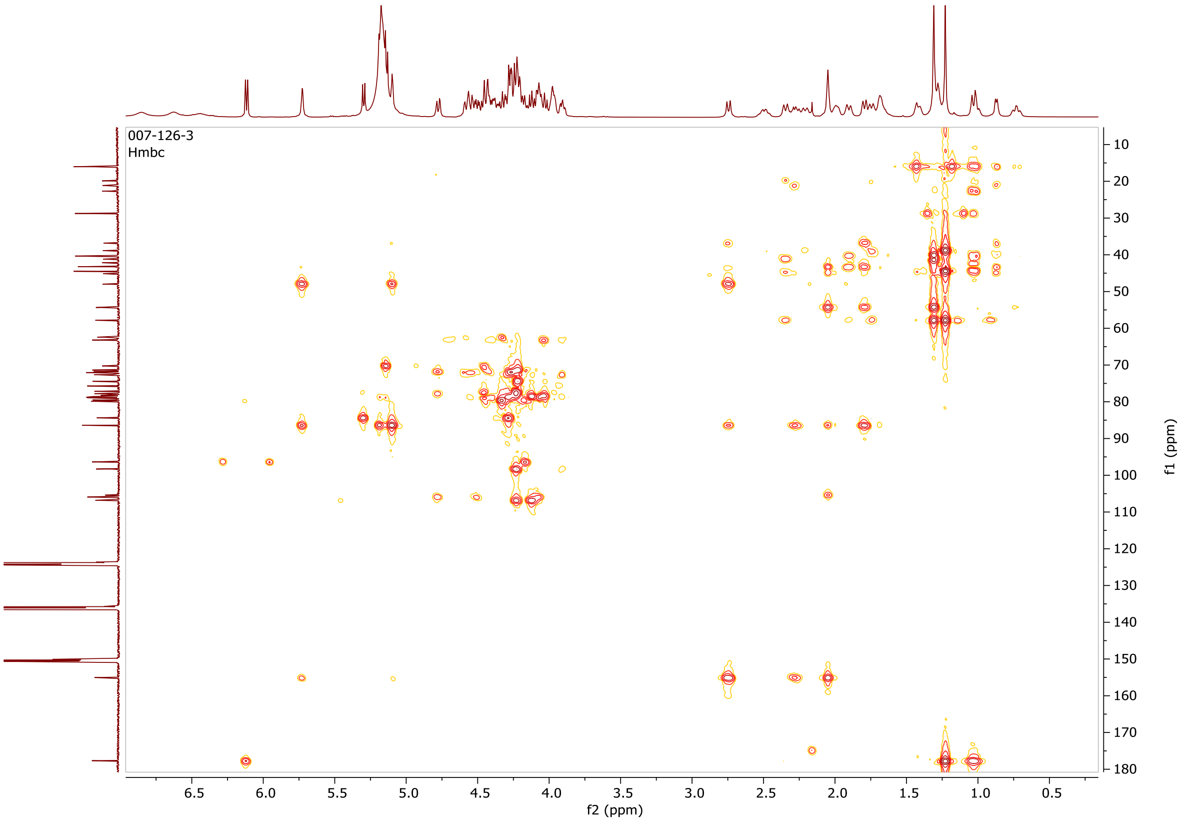

Figure 7. HMBC spectrum of compound **1**.

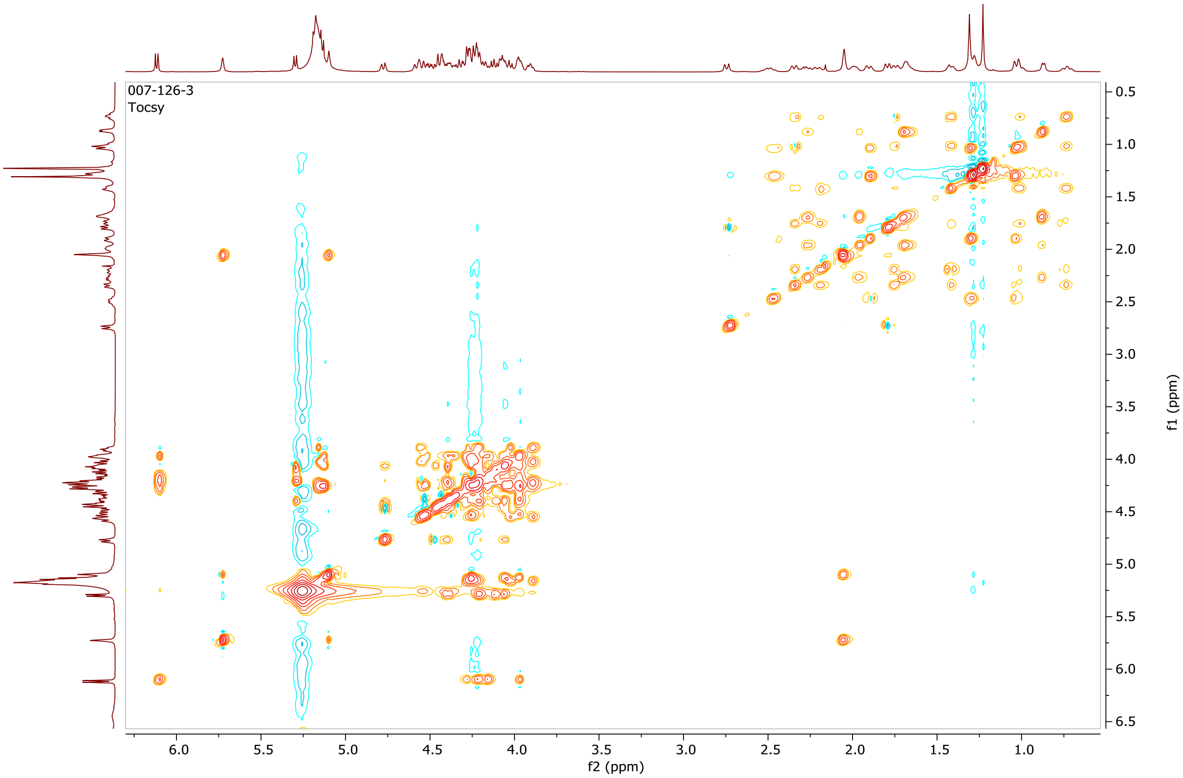

Figure 8. TOCSY spectrum of compound **1**.

Rebaudioside Z_1
(compound 2)

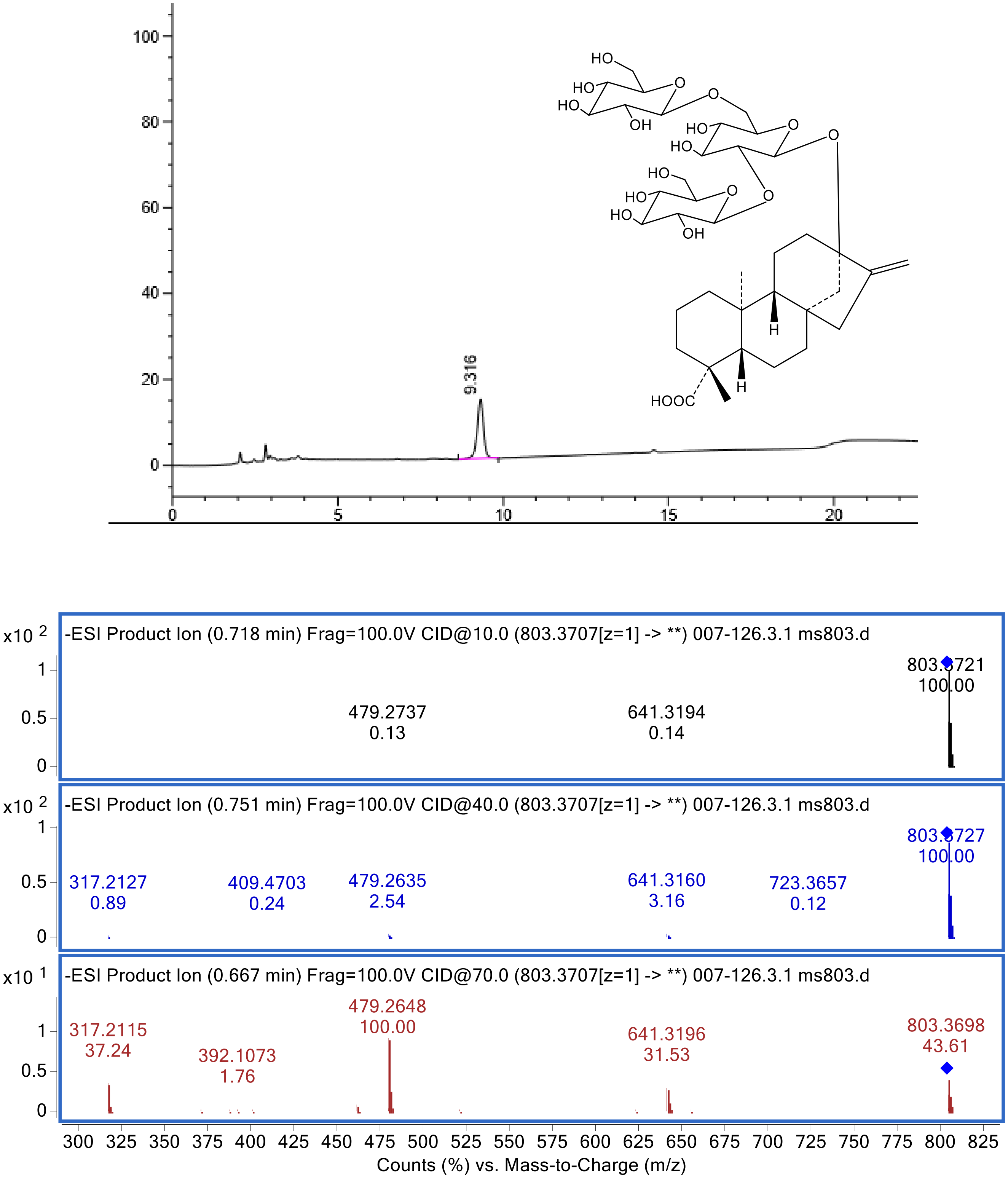

Figure 9. RP-C18 HPLC chromatogram**,** chemical structure and MS/MS spectrum of compound **2**.

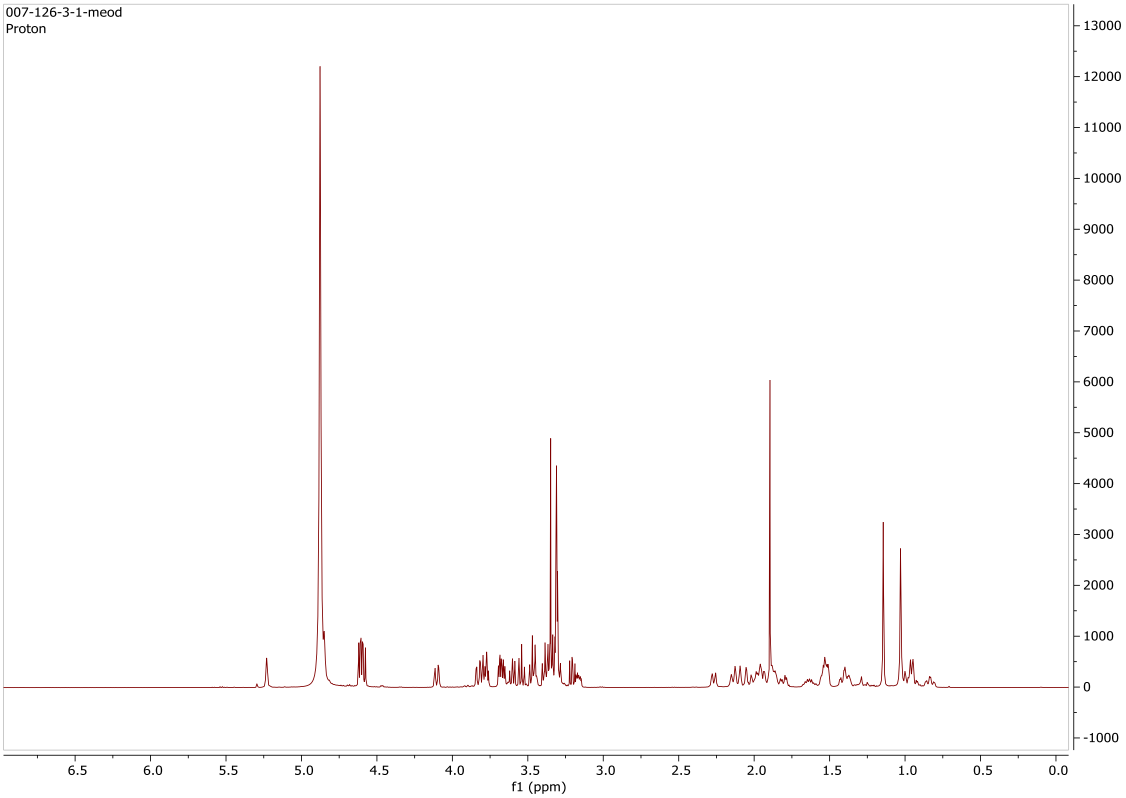

Figure 10. ^{1}H NMR spectrum of compound **2**.

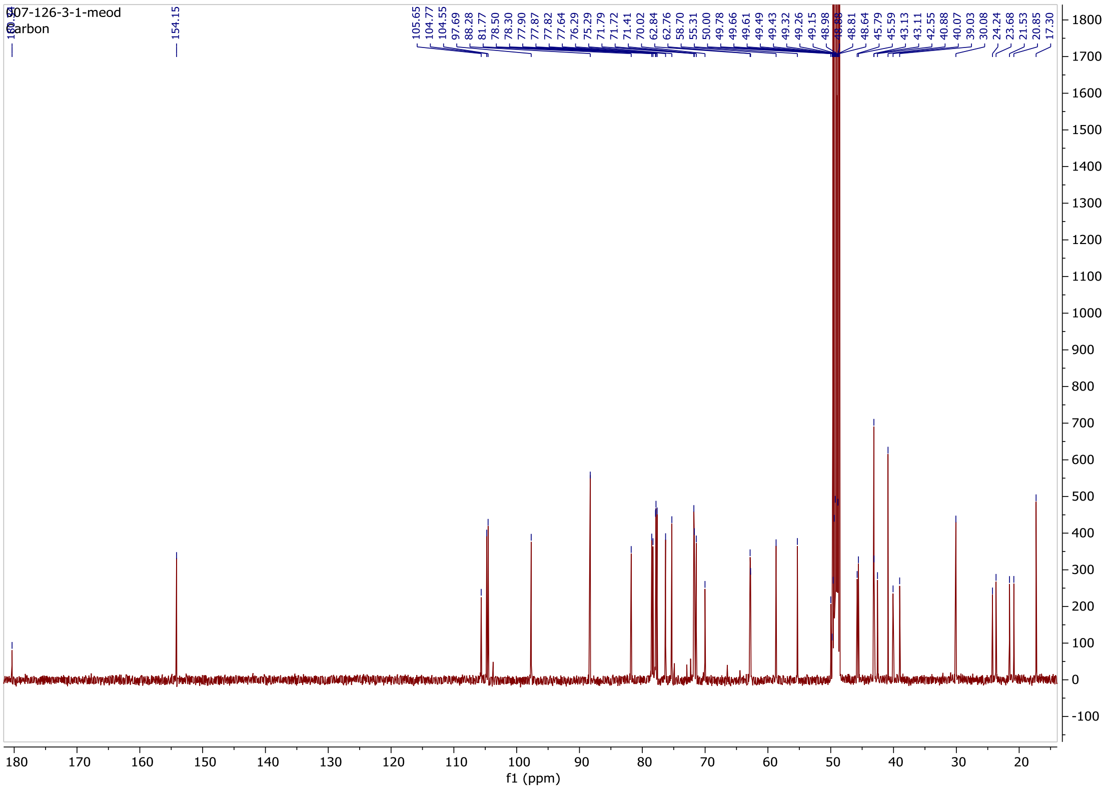

Figure 11. ^{13} C NMR spectrum of compound **2**.

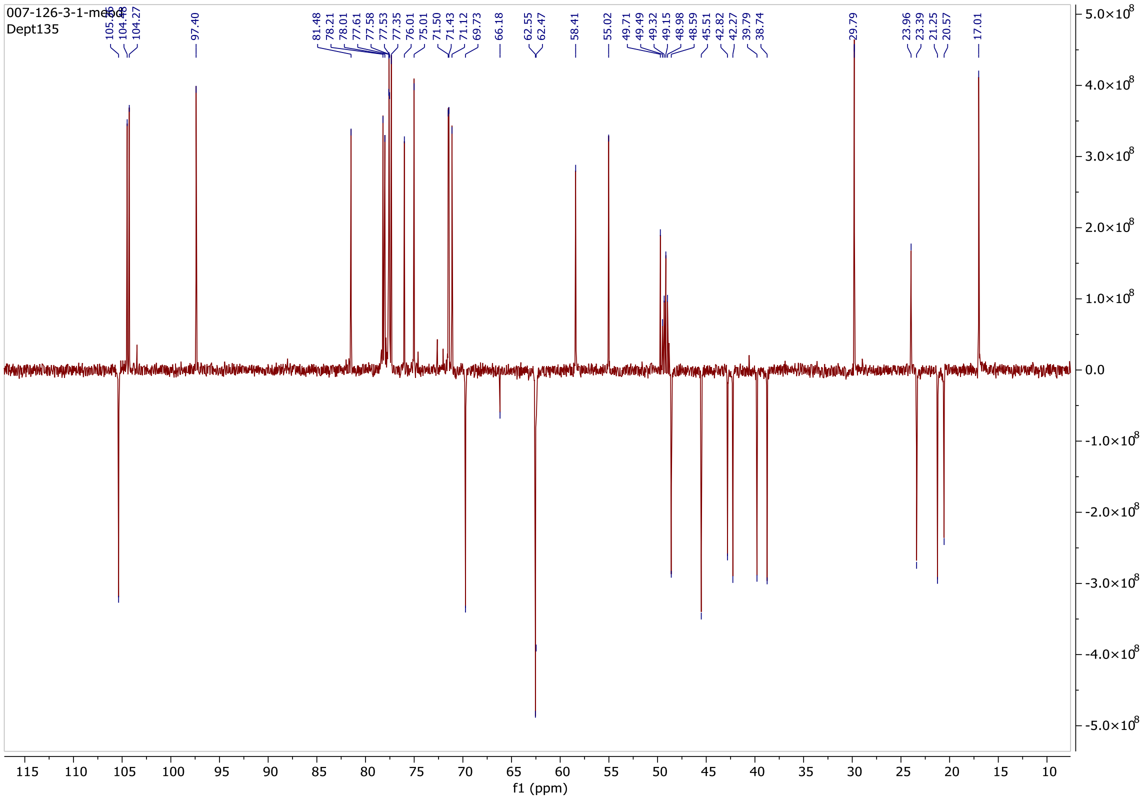

Figure 12. DEPT 135 spectrum of compound **2**.

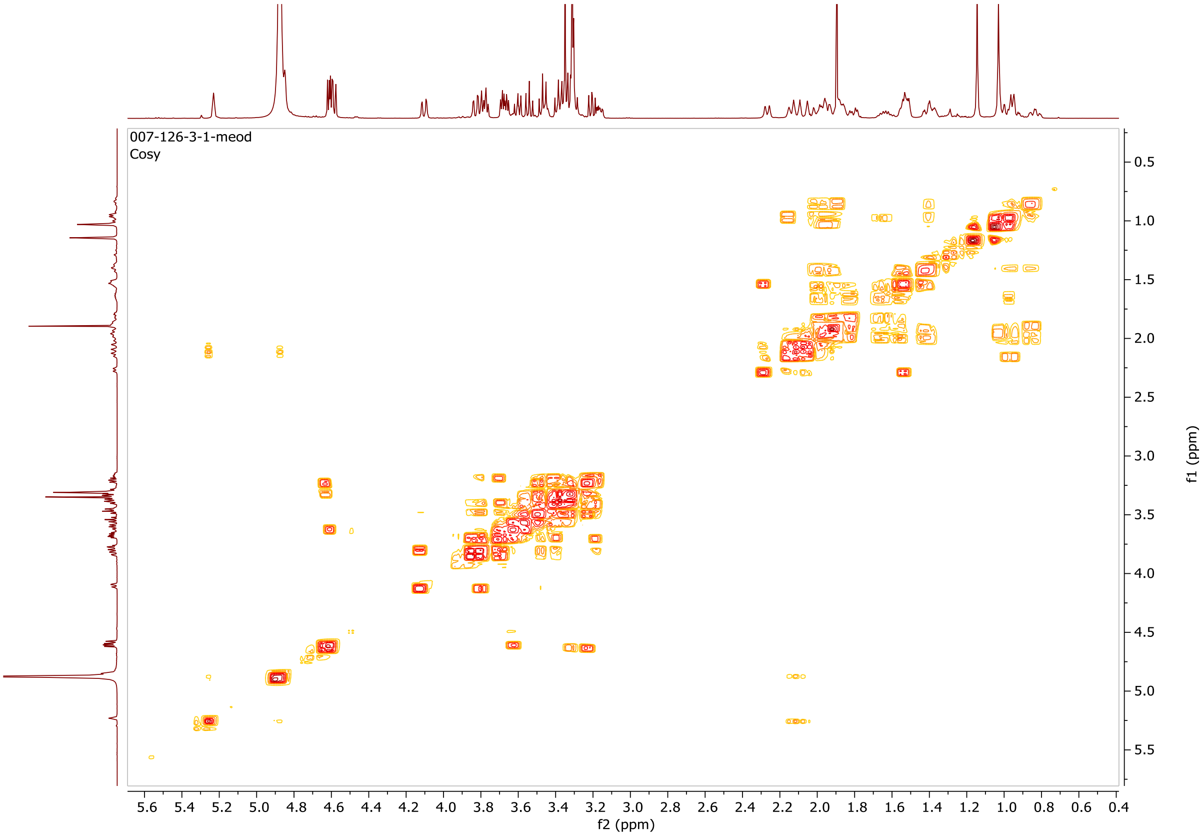

Figure 13. DQCOSY spectrum of compound **2**.

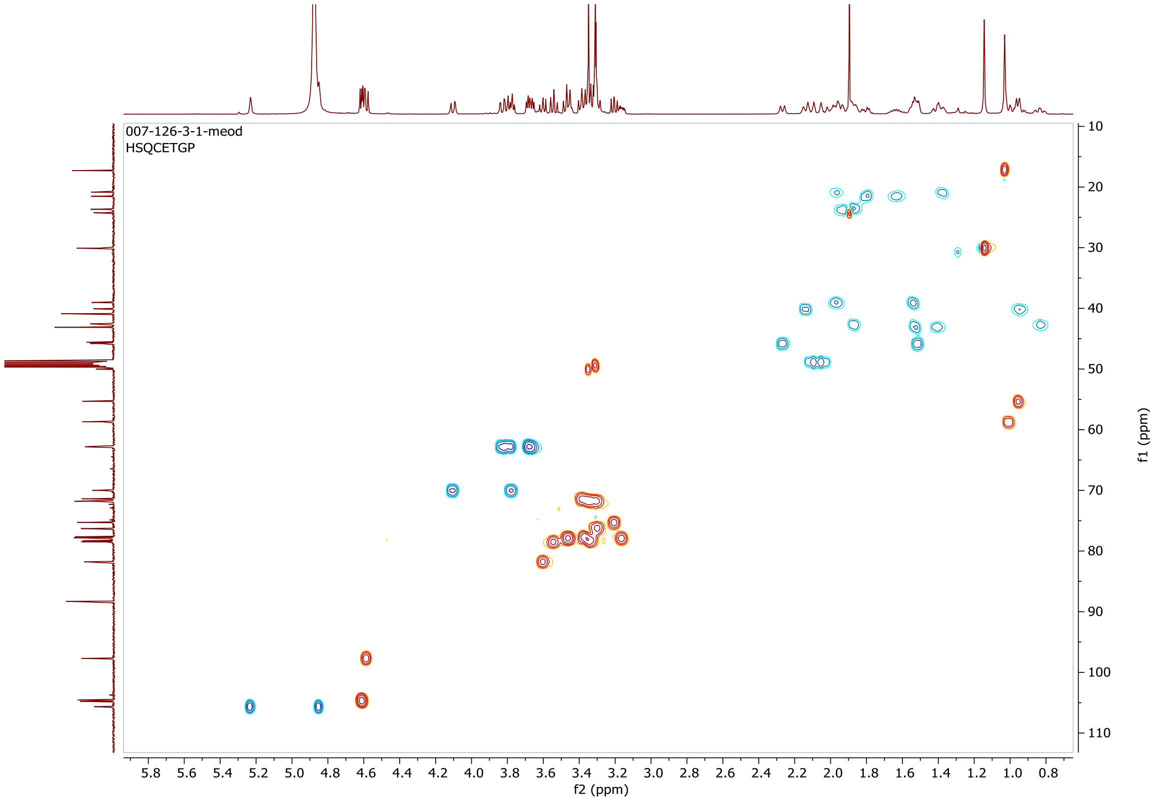

Figure 14. HSQC spectrum of compound **2**.

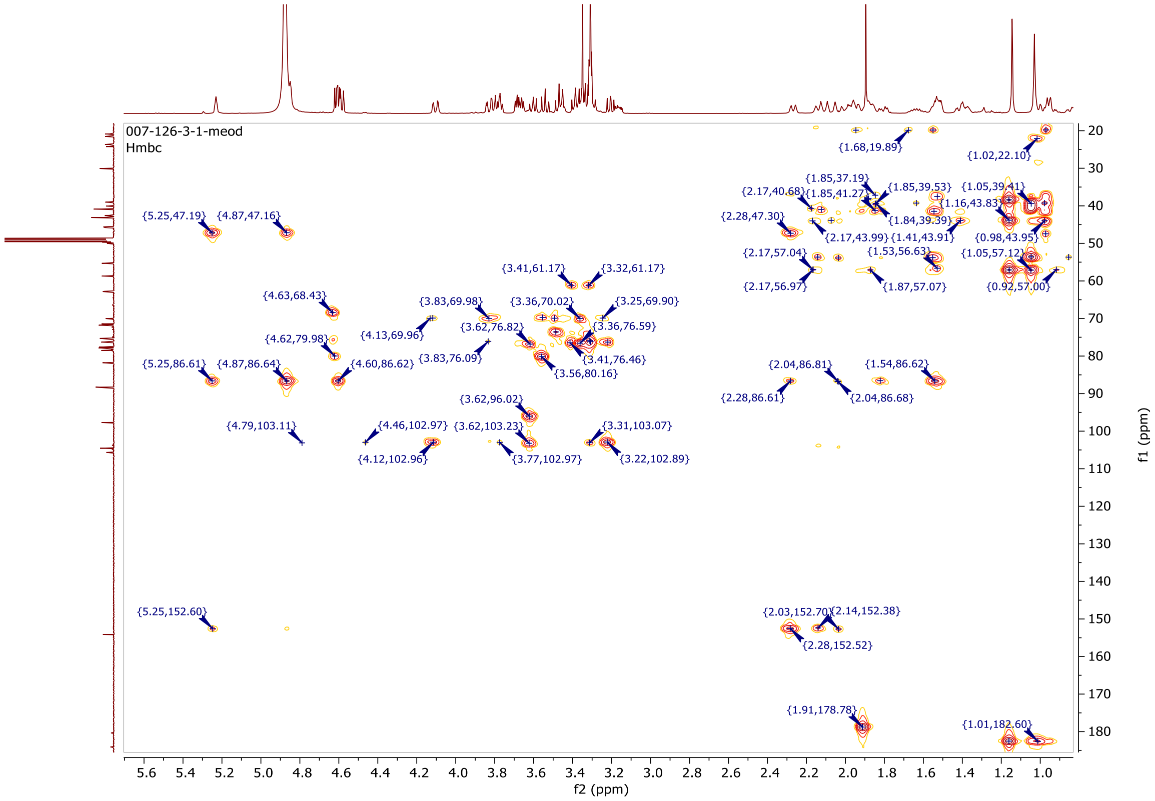

Figure 15. HMBC spectrum of compound **2**.

13-[(2-*O*-β-D-glucopyranosyl-3-*O*-β-D-glucopyranosyl-β-D-glucopyranosyl) oxy]*ent*-hydroxyatis-16-en-19-oic acid -β-D-glucopyranosy ester (compound 3)

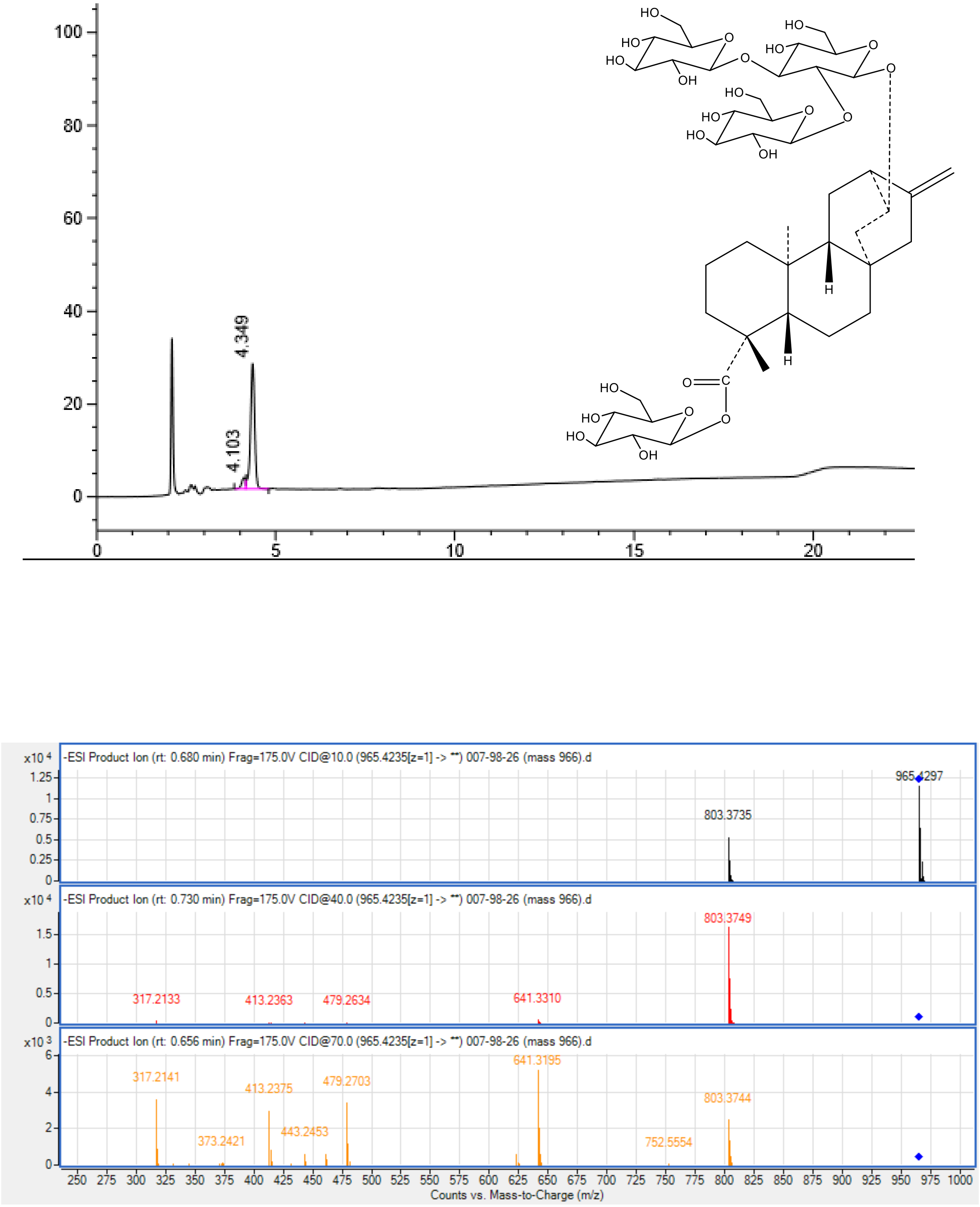

Figure 16. RP-C18 HPLC chromatogram**,** chemical structure and MS/MS spectrum of compound **3**.

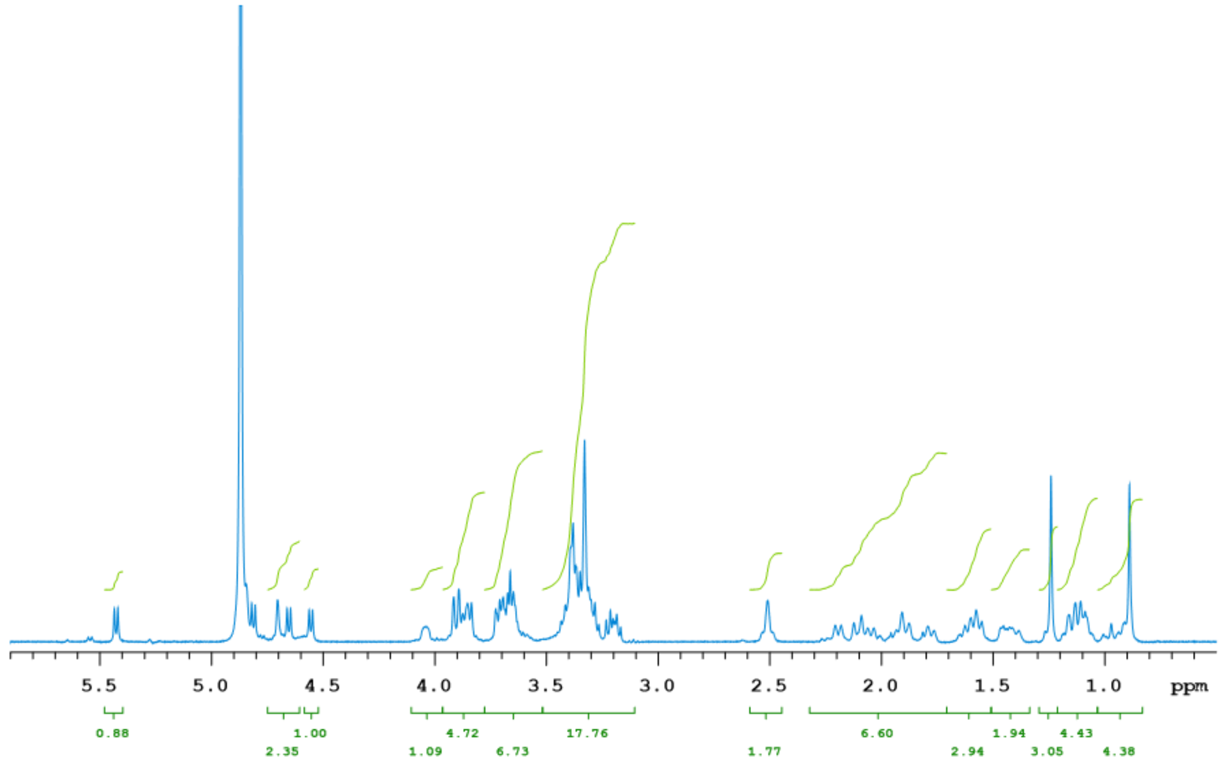

Figure 17. ^{1}H NMR spectrum of compound **3**.

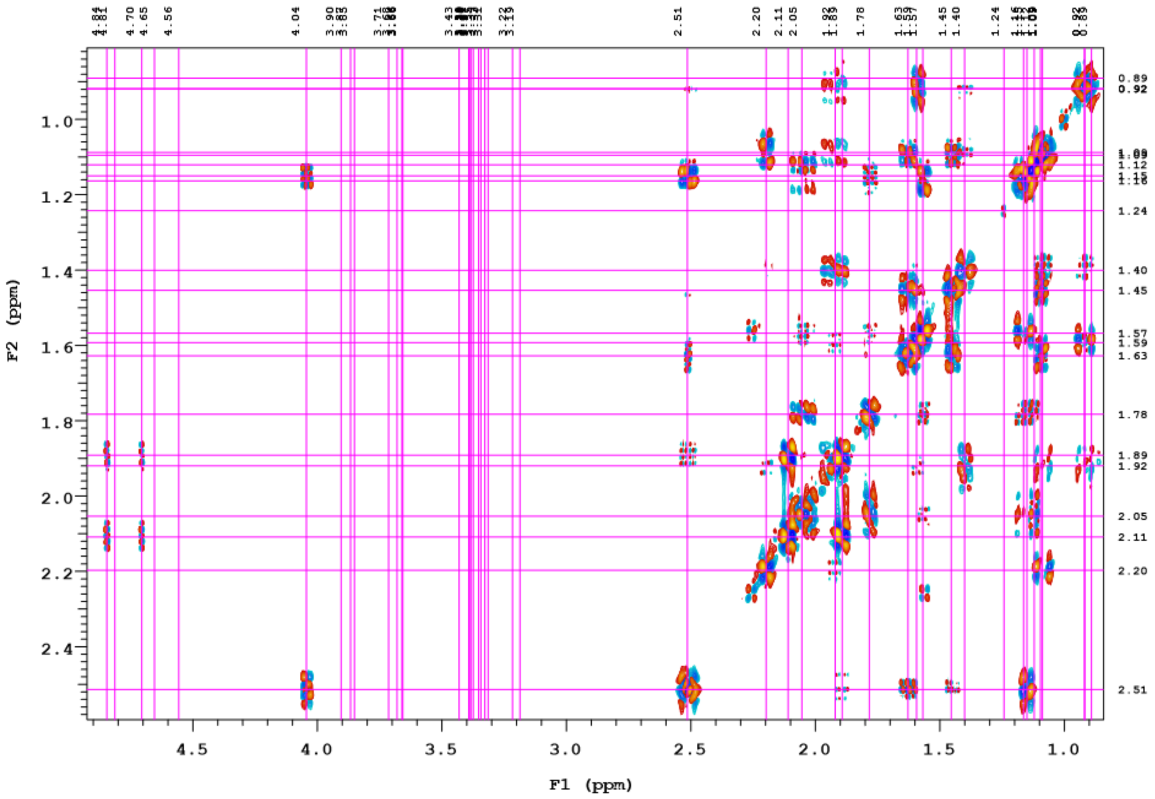

Figure 18. DQCOSY spectrum of compound 3.

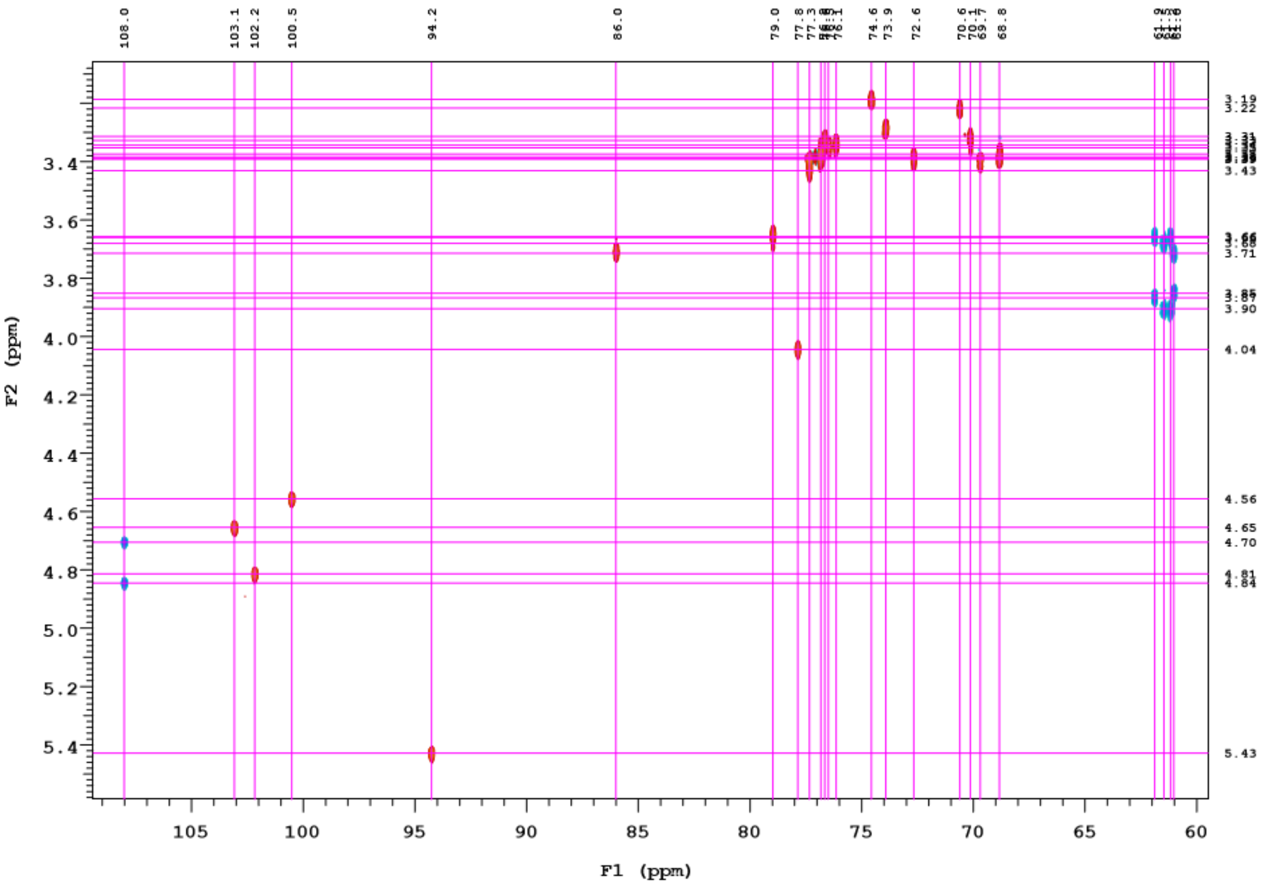

Figure 19. HSQC spectrum of compound 3 from 60 - 110 ppm.

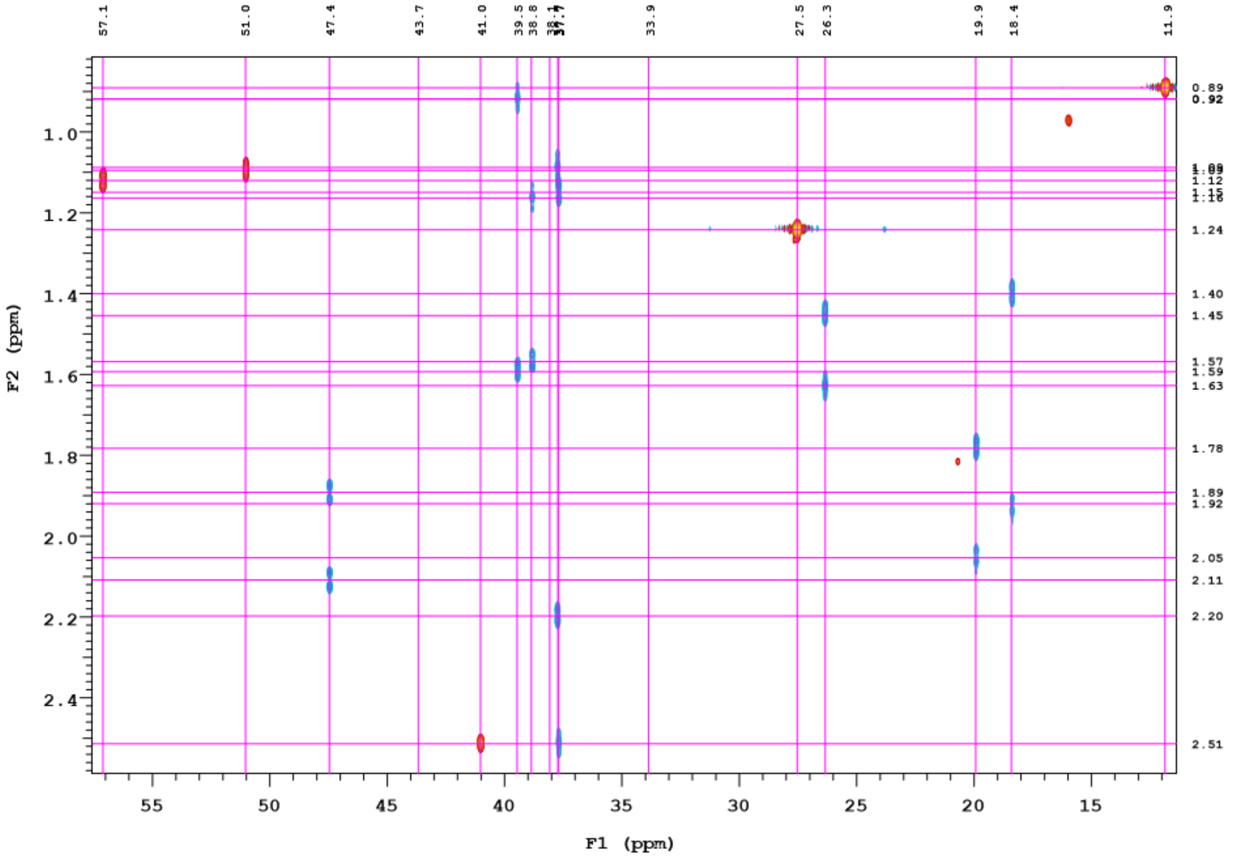

Figure 20. HSQC spectrum of compound 3 from 10 – 60 ppm.

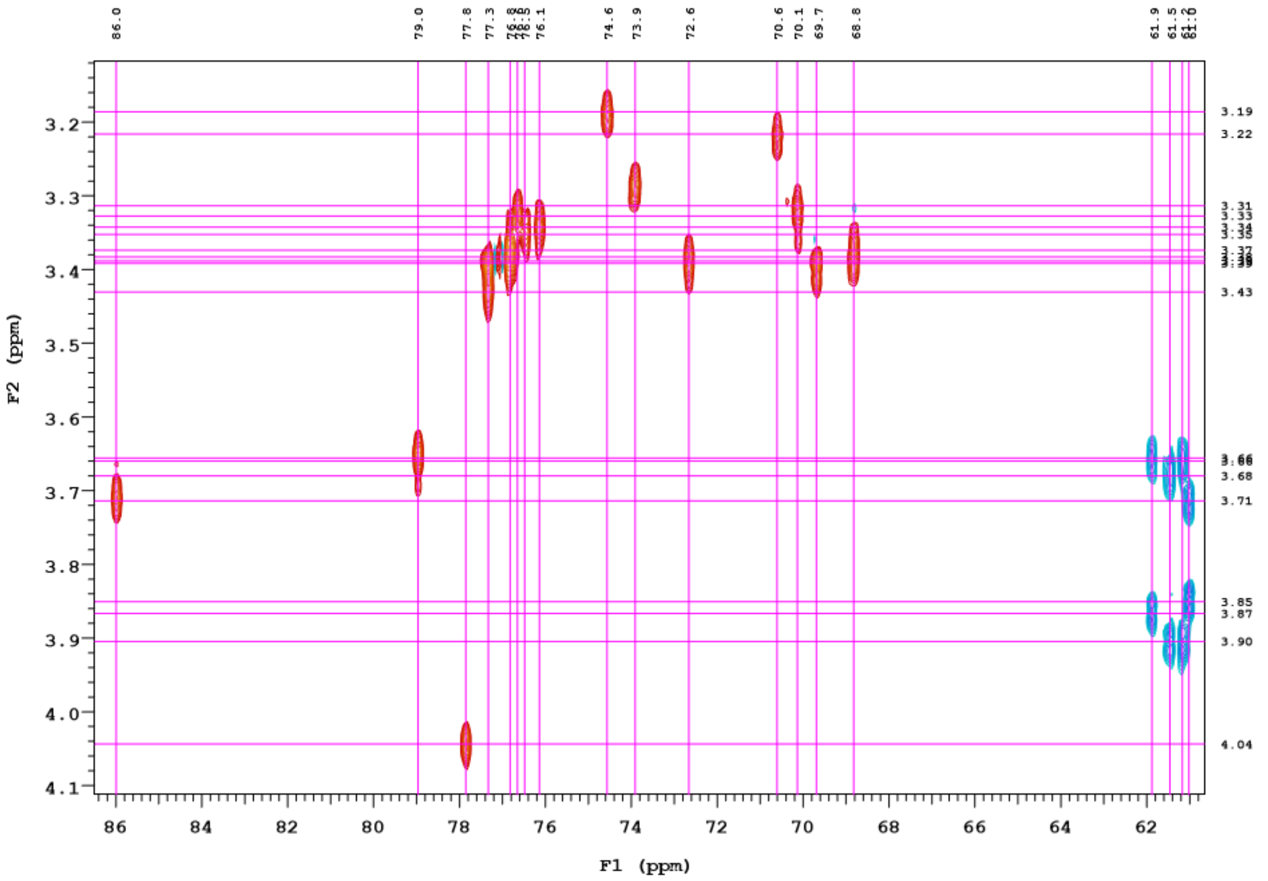

Figure 21. HSQC spectrum of compound 3 from 60 – 88 ppm.

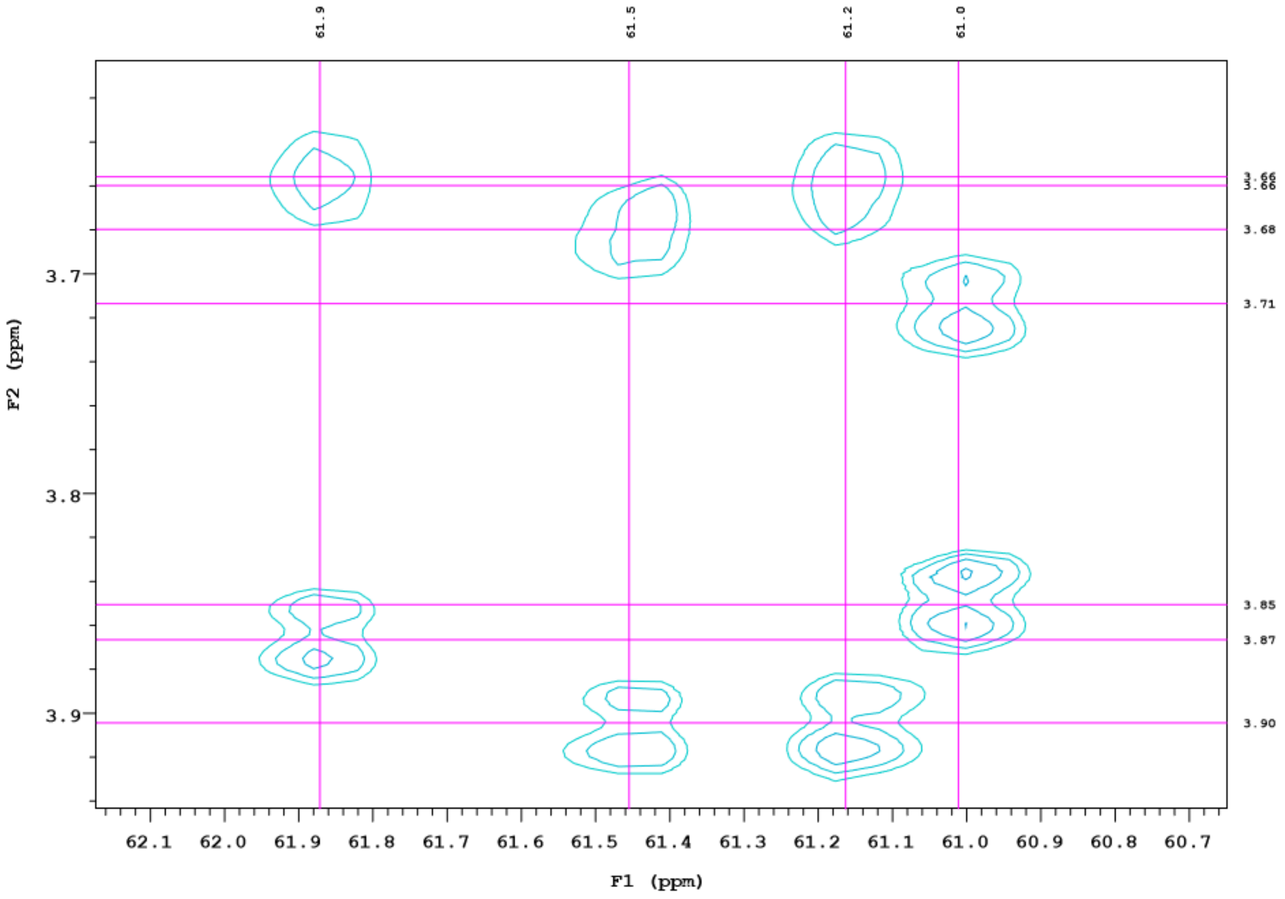

Figure 22. HSQC spectrum of compound 3 from 60 – 63 ppm.

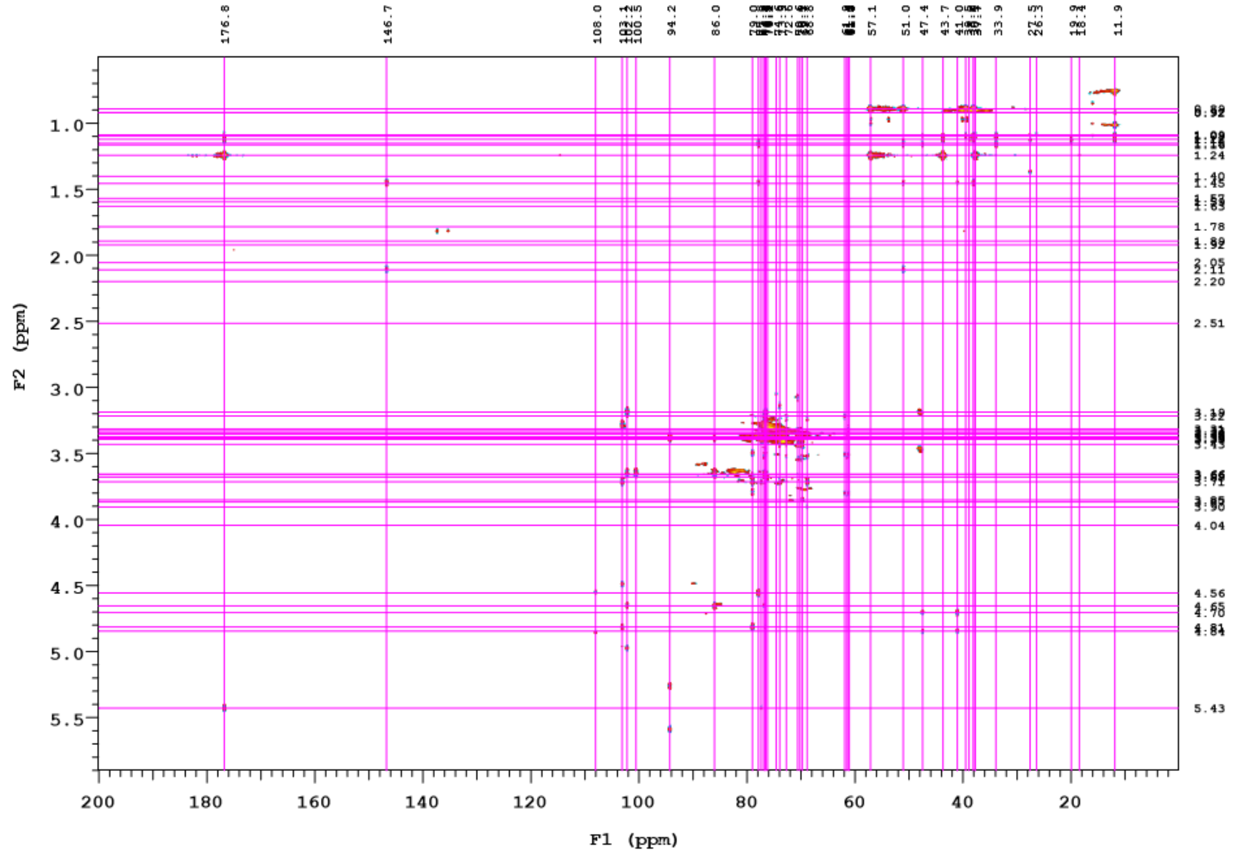

Figure 23. HMBC spectrum of compound 3.

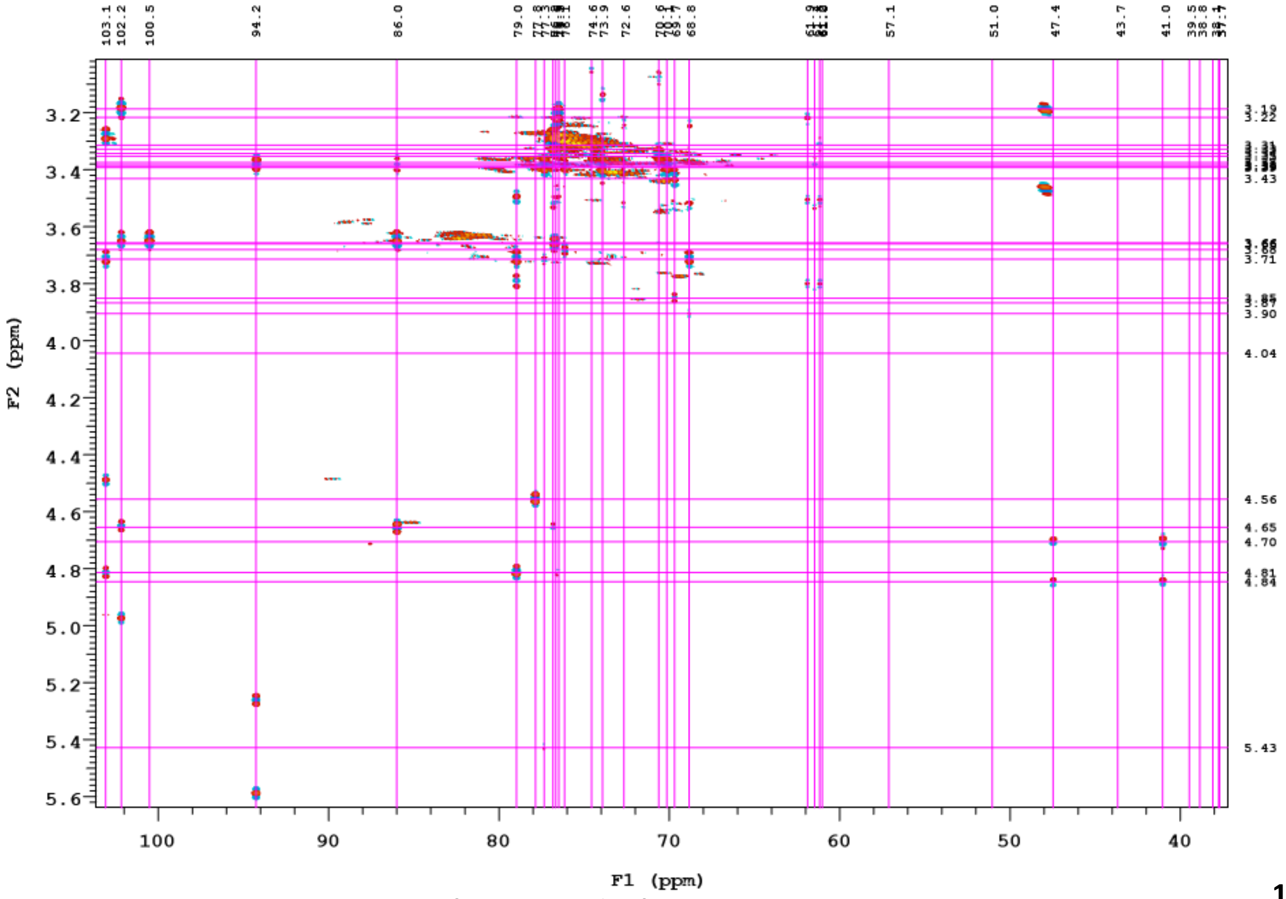

Figure 24. HMBC spectrum of compound 3 from 35 – 105 ppm.

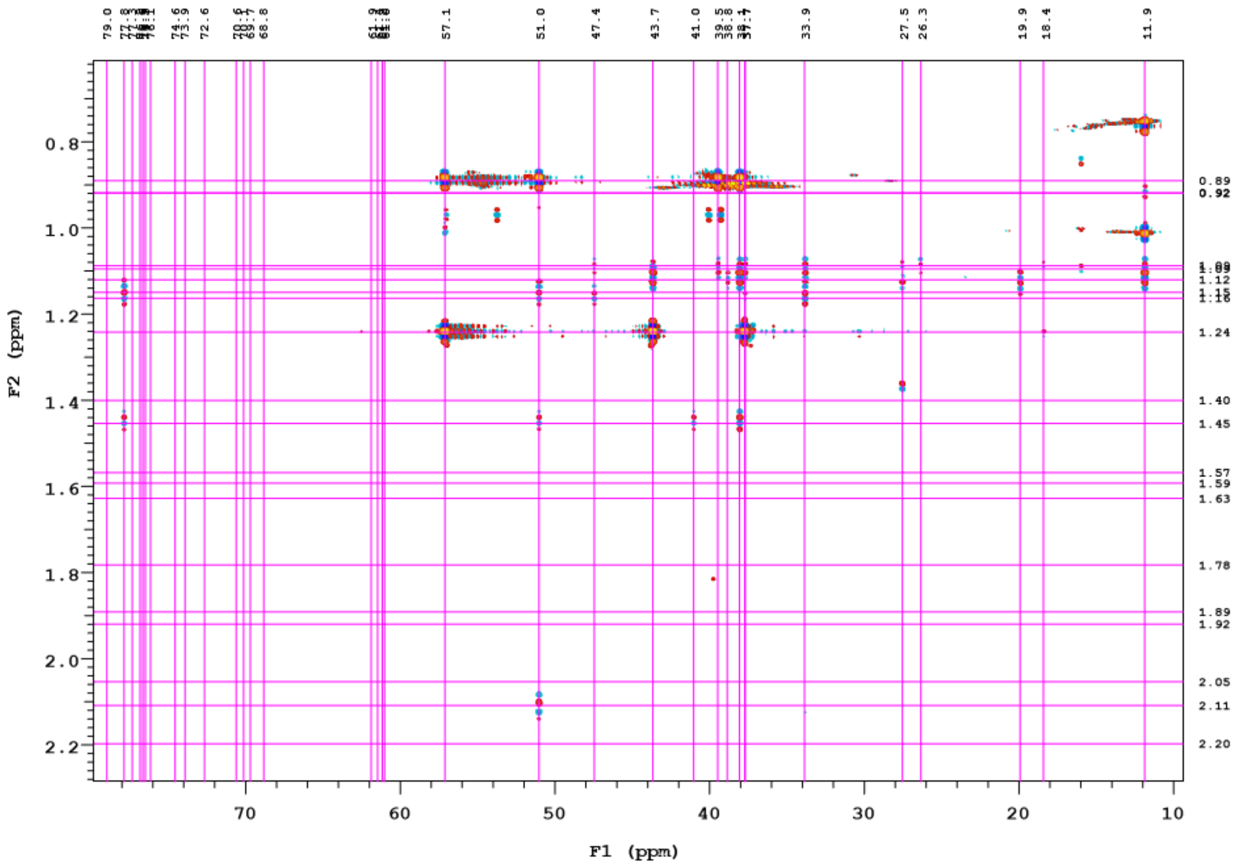

Figure 25. HMBC spectrum of compound 3 from 10 – 80 ppm.

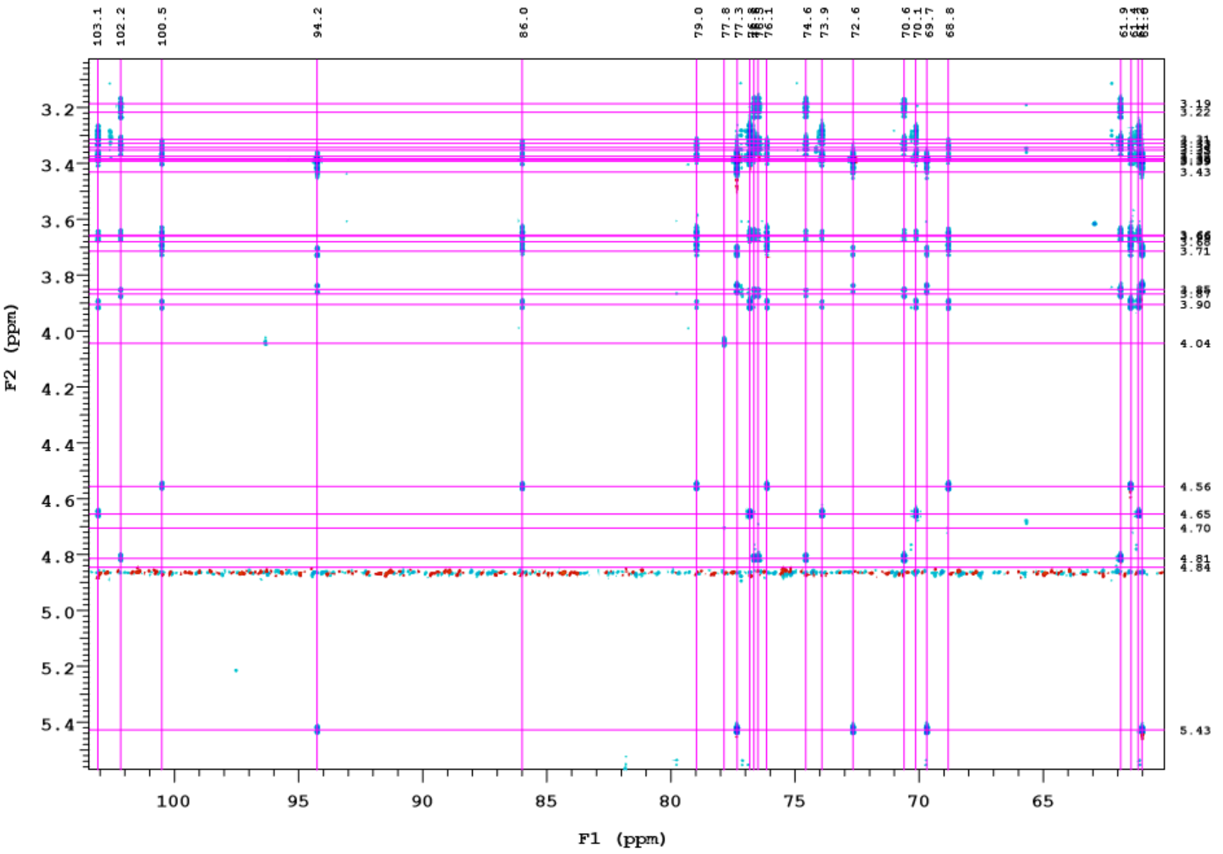

Figure 26. HMBC spectrum of compound 3 from 60 – 105 ppm.

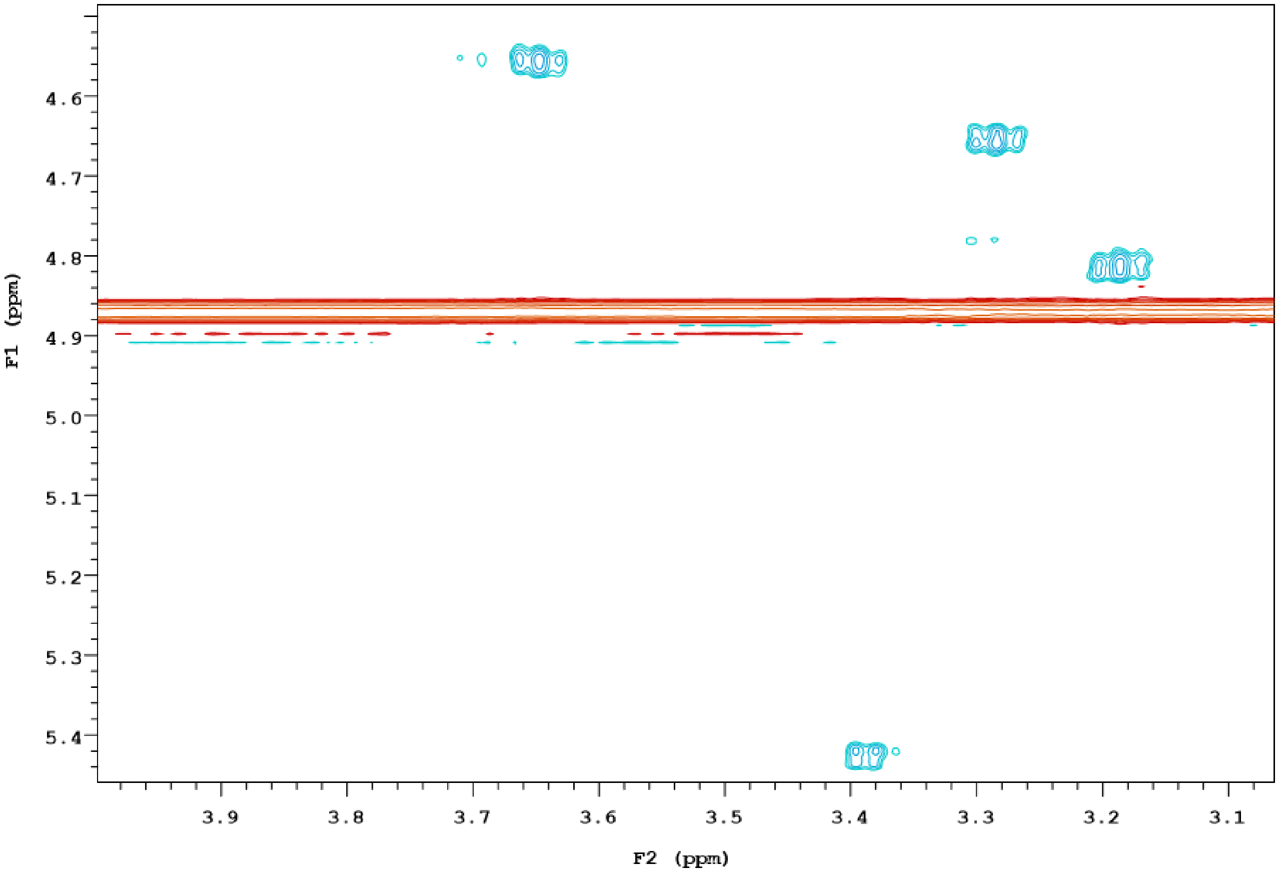

Figure 27. ^{1}H-^{1}H band-selective TOCSY spectrum of compound 3 with a mixing time of 50 ms.

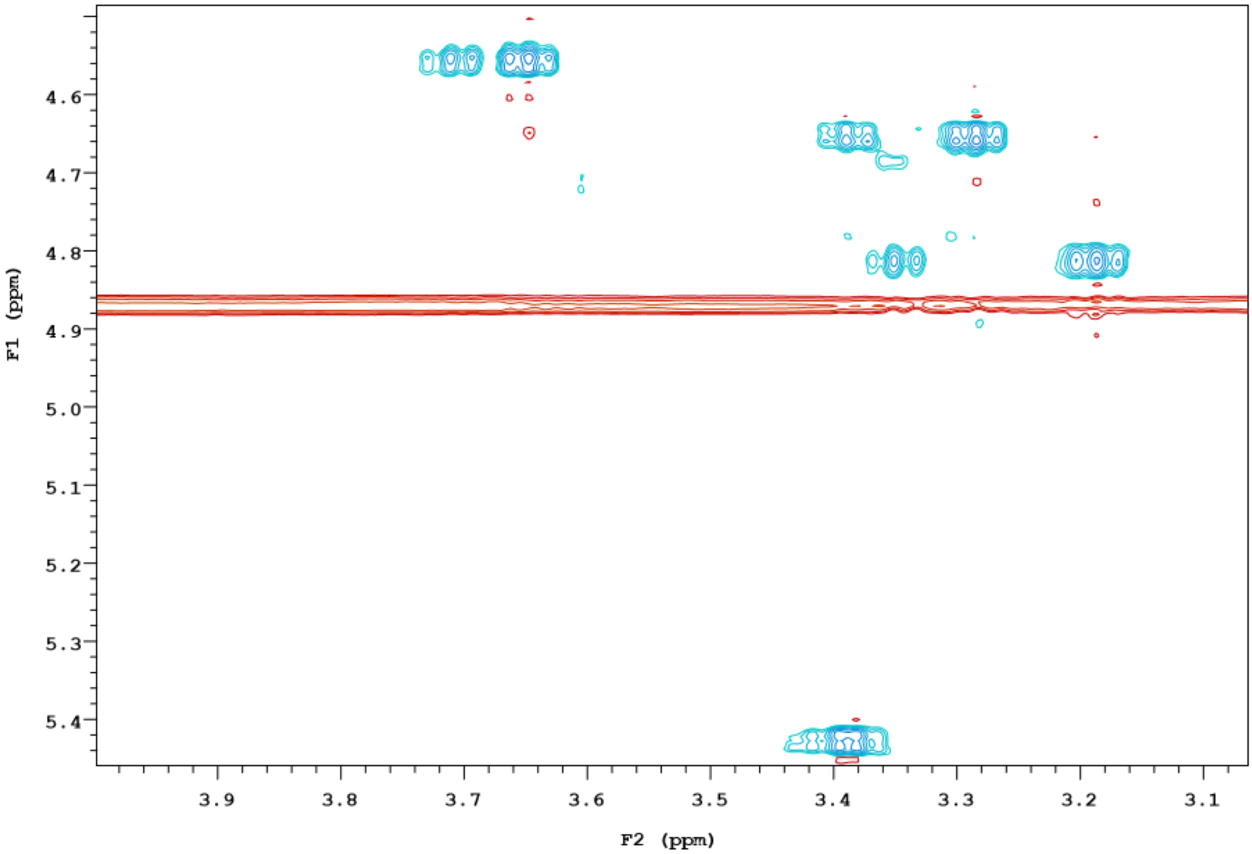

Figure 28. ^{1}H-^{1}H band-selective TOCSY spectrum of compound 3 with a mixing time of 70 ms.

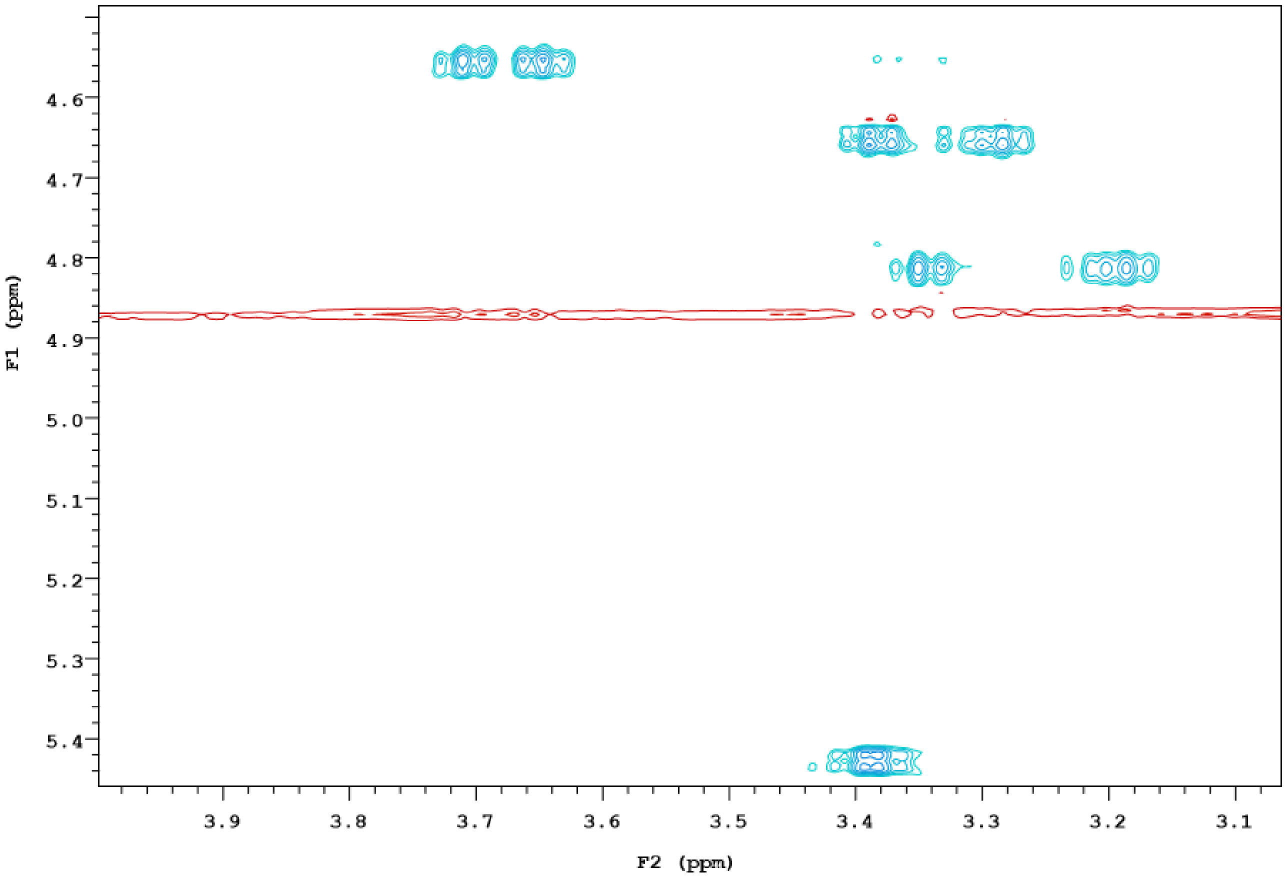

Figure 29. ^{1}H-^{1}H band-selective TOCSY spectrum of compound 3 with a mixing time of 100 ms.

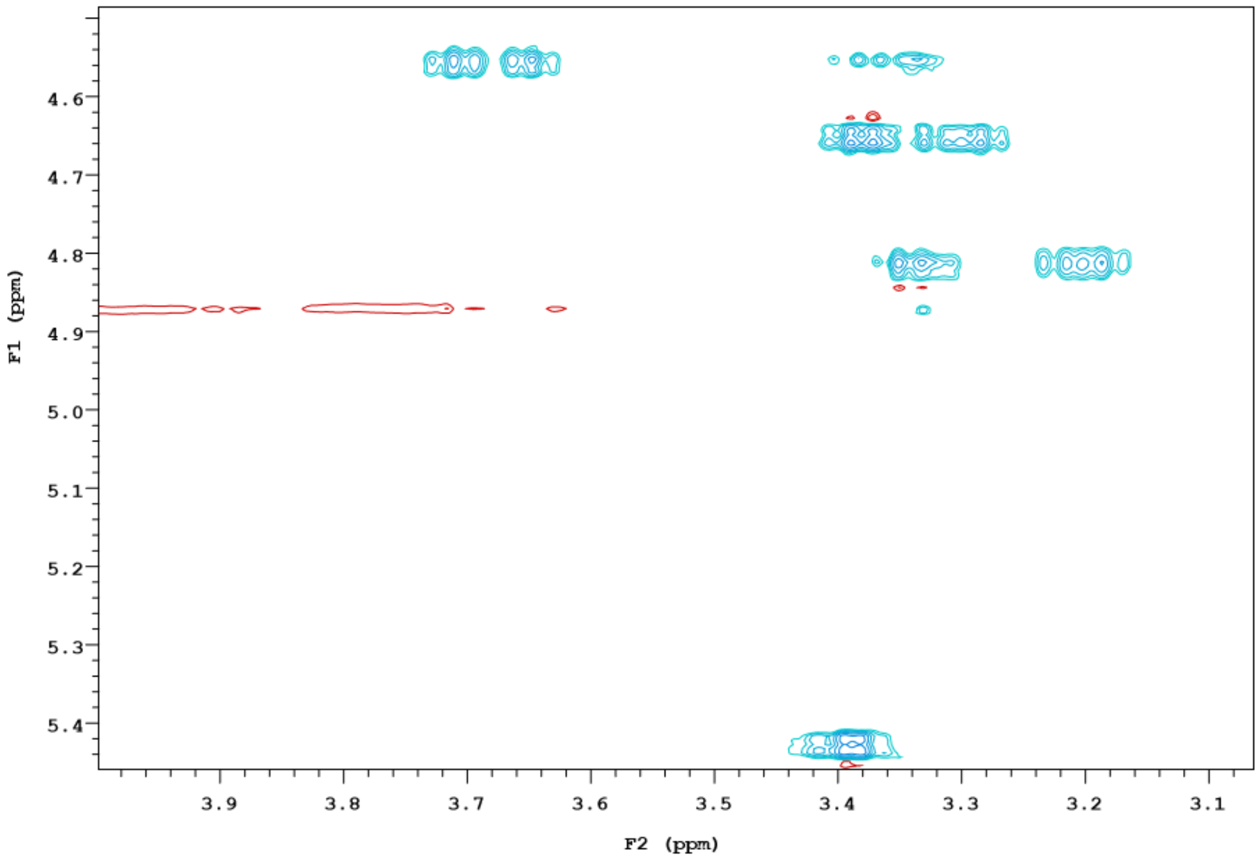

Figure 30. ^{1}H-^{1}H band-selective TOCSY spectrum of compound 3 with a mixing time of 150 ms.

13-[(2-*O*-β-D-glucopyranosyl-3-*O*-β-D-glucopyranosyl-β-D-glucopyranosyl) oxy]*ent*-hydroxyatis-16-en-19-oic acid (compound 4)

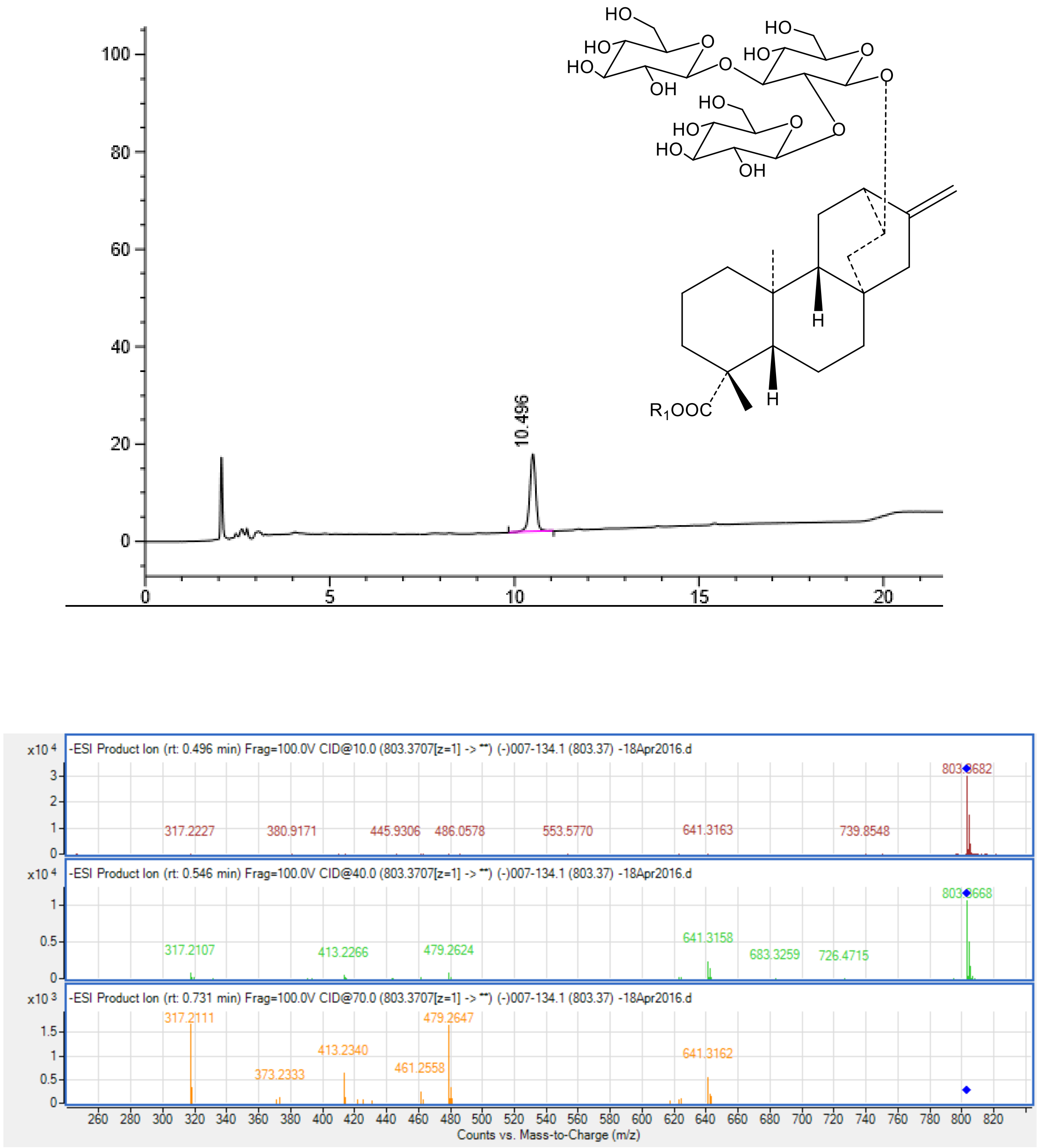

Figure 31. RP-C18 HPLC chromatogram**,** chemical structure and MS/MS spectrum of compound **4**.

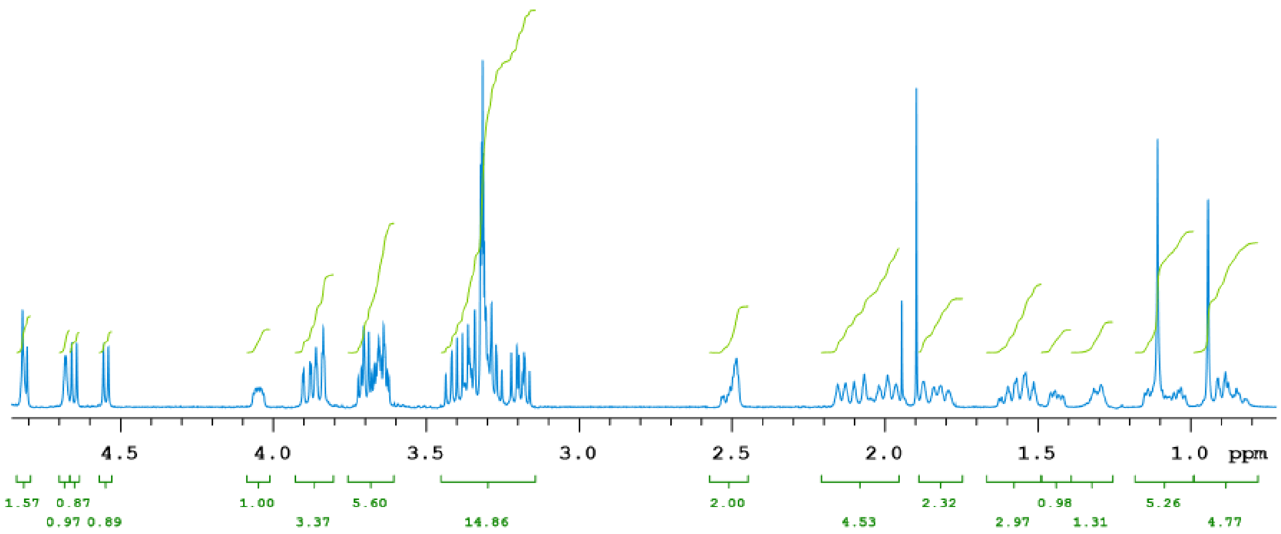

Figure 32. ^{1}H NMR spectrum of compound **4**.

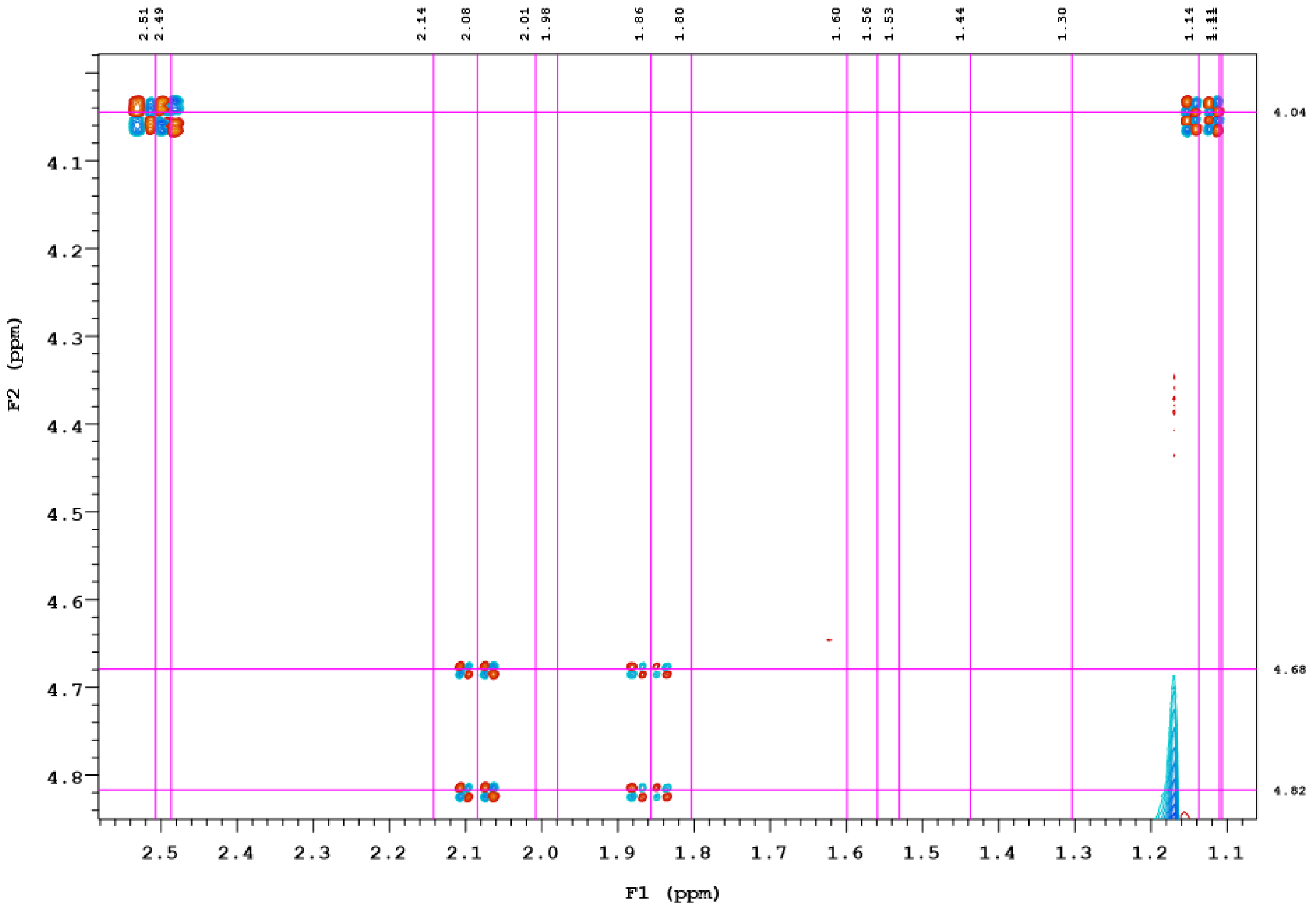

Figure 33. DQCOSY spectrum of compound 4.

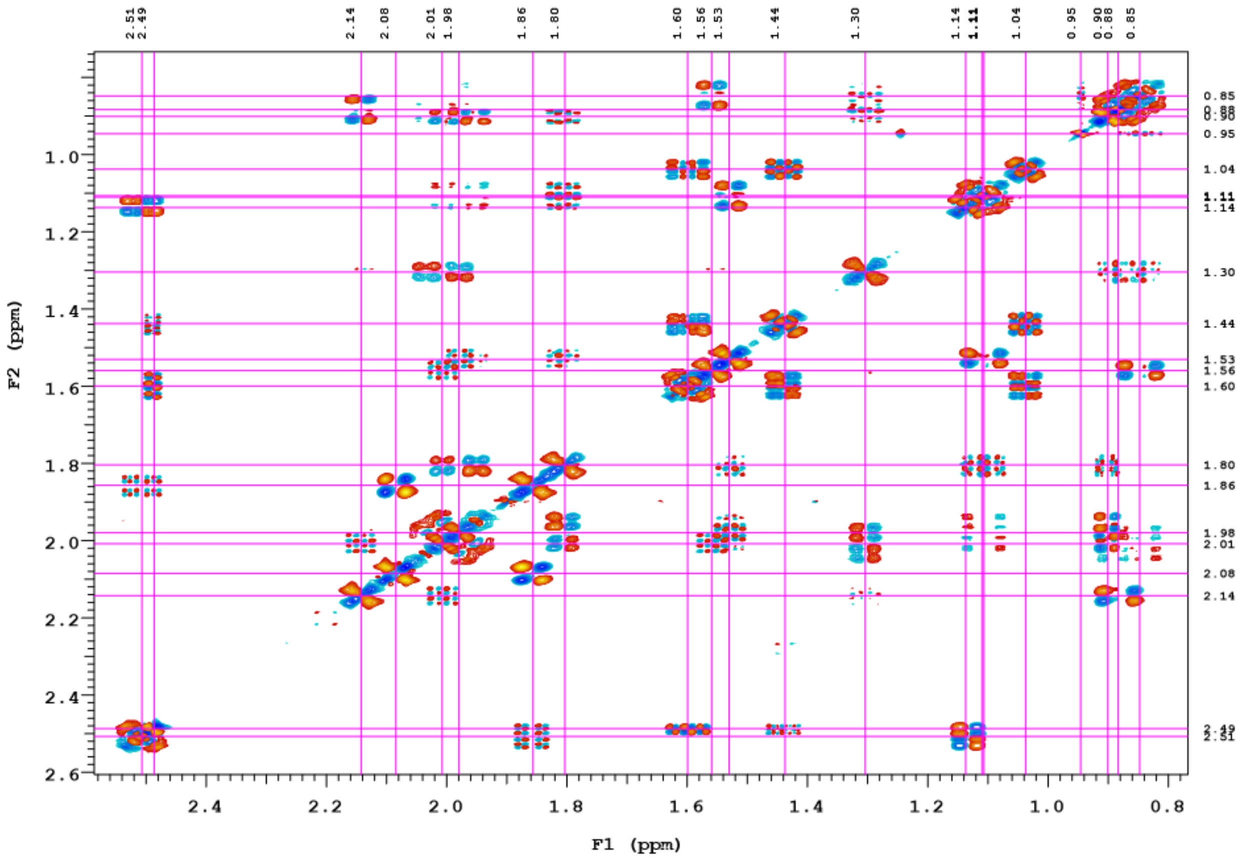

Figure 34. DQCOSY spectrum of compound 4.

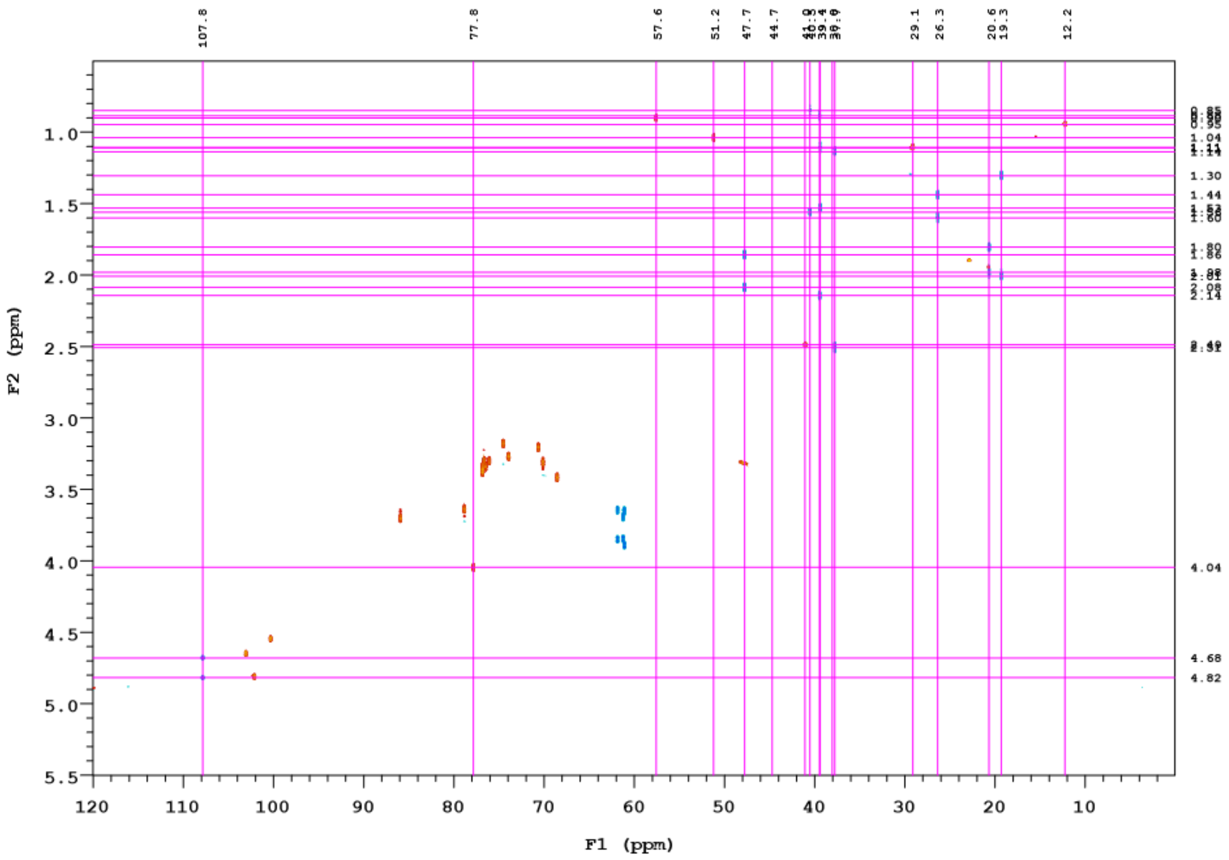

Figure 35. HSQC spectrum of compound 4 from 0 - 120 ppm.

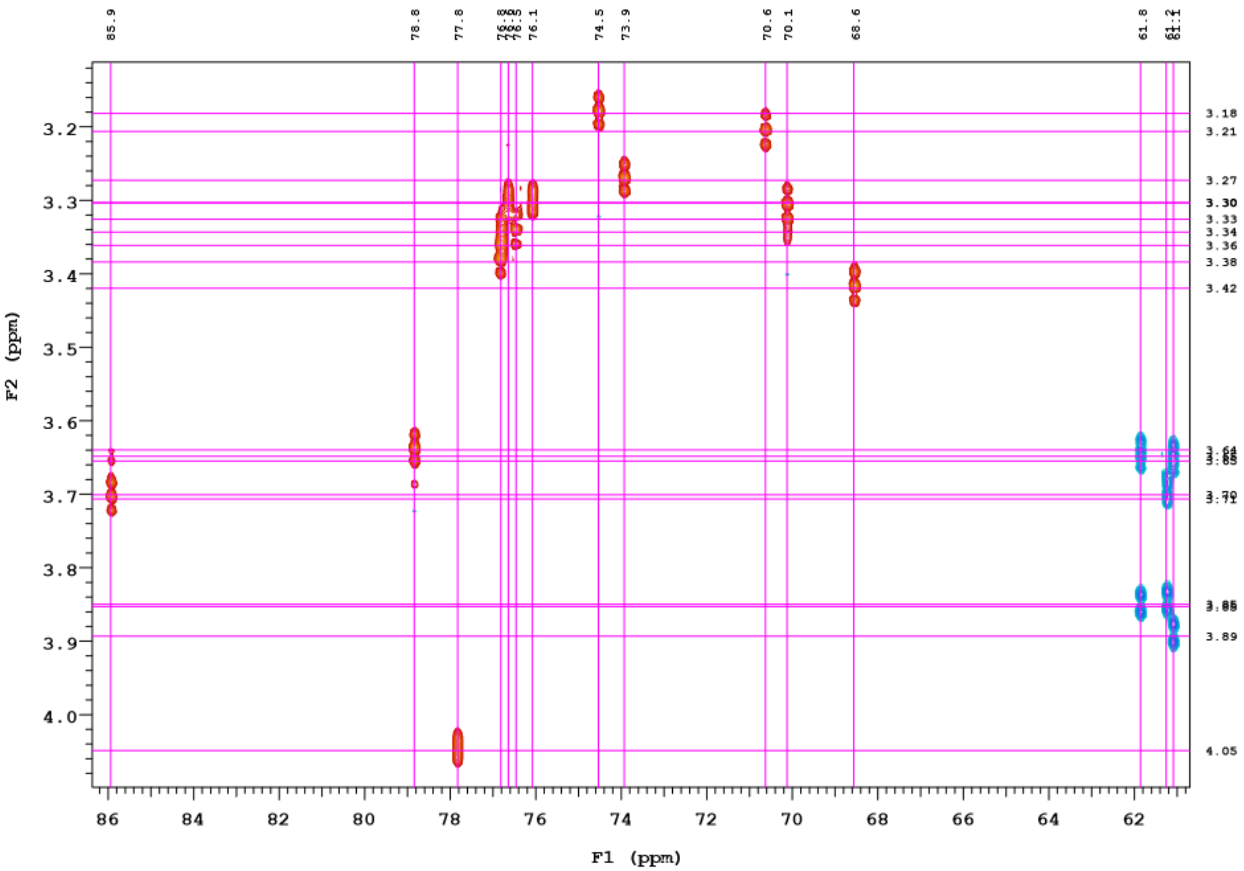

Figure 36. HSQC spectrum of compound 4 from 60 - 86 ppm.

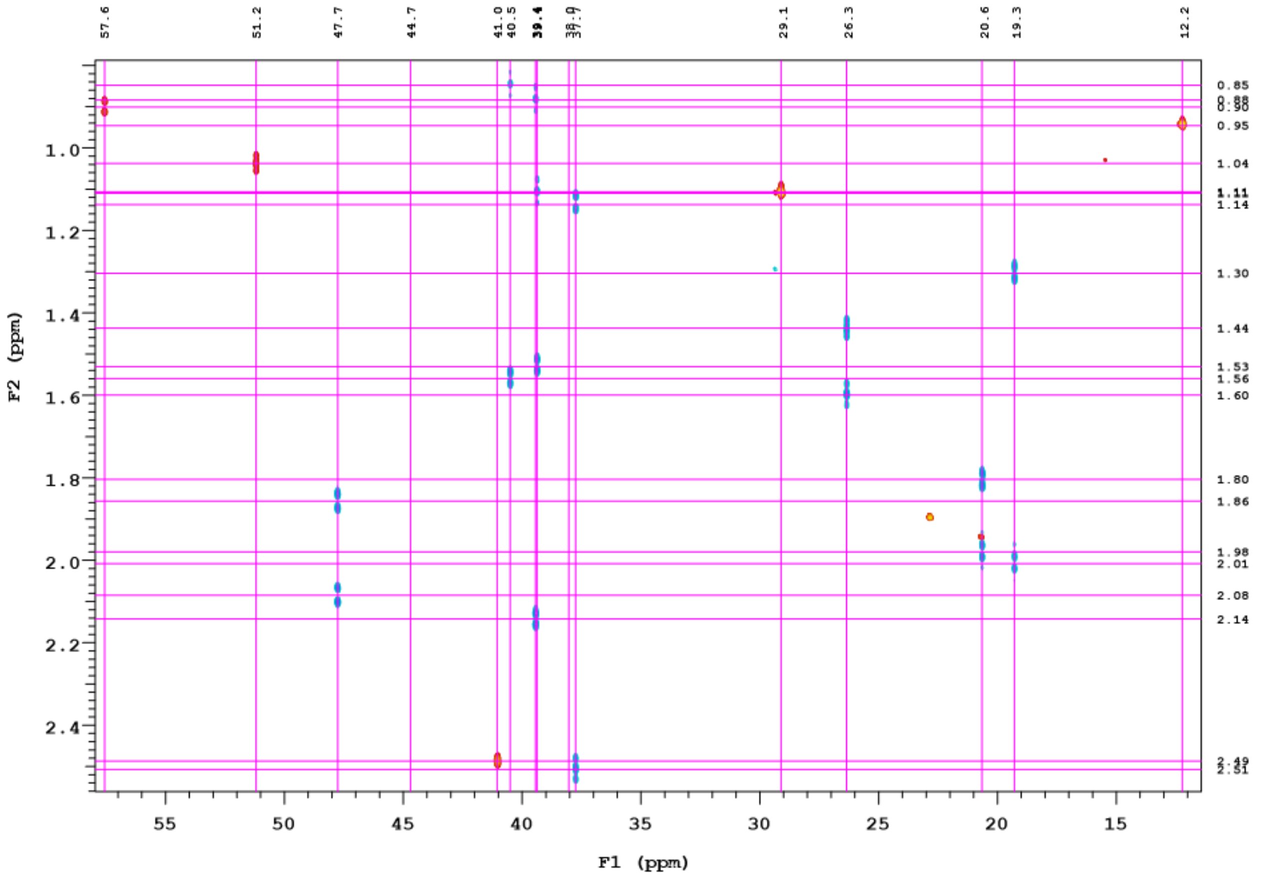

Figure 37. HSQC spectrum of compound 4 from 10 - 60 ppm.

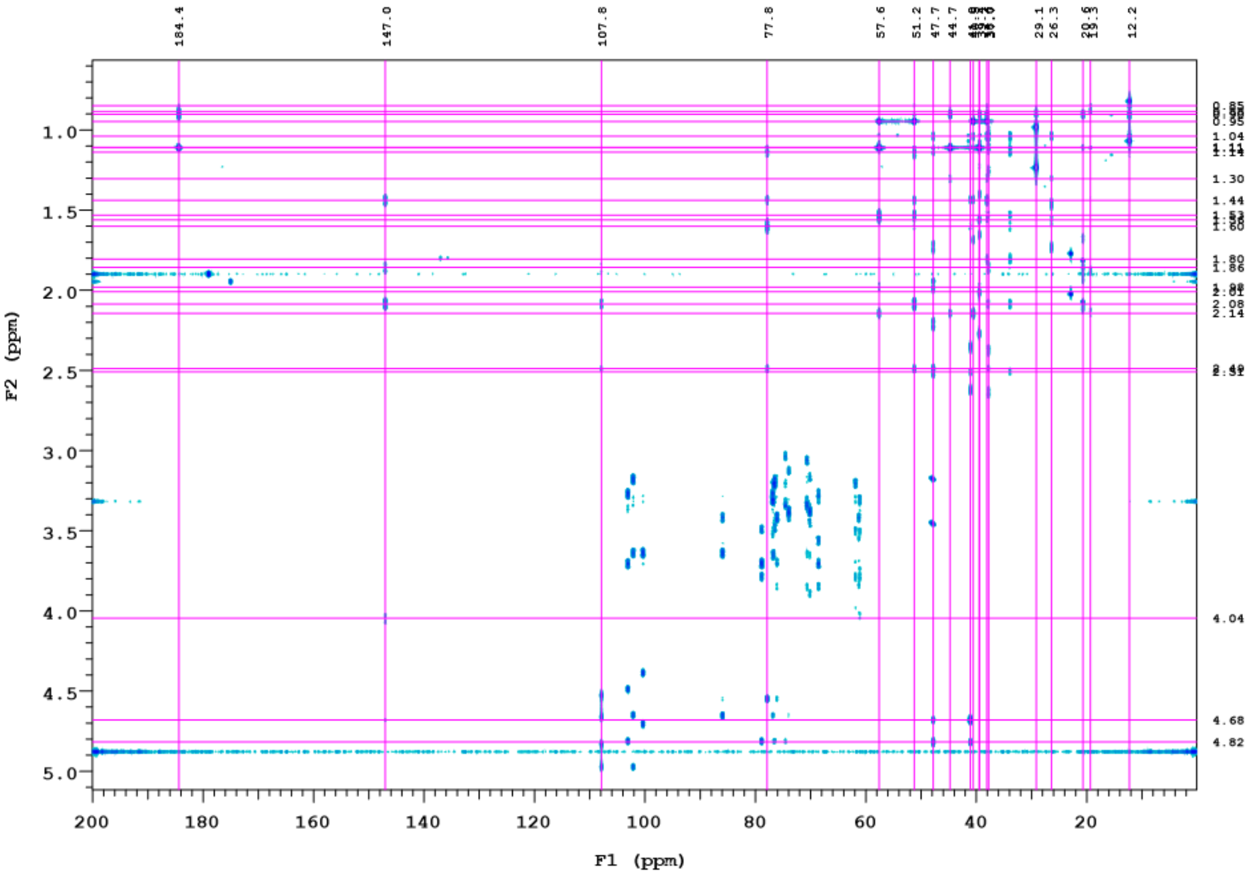

Figure 38. HMBC spectrum of compound 4 from 10 - 200 ppm.

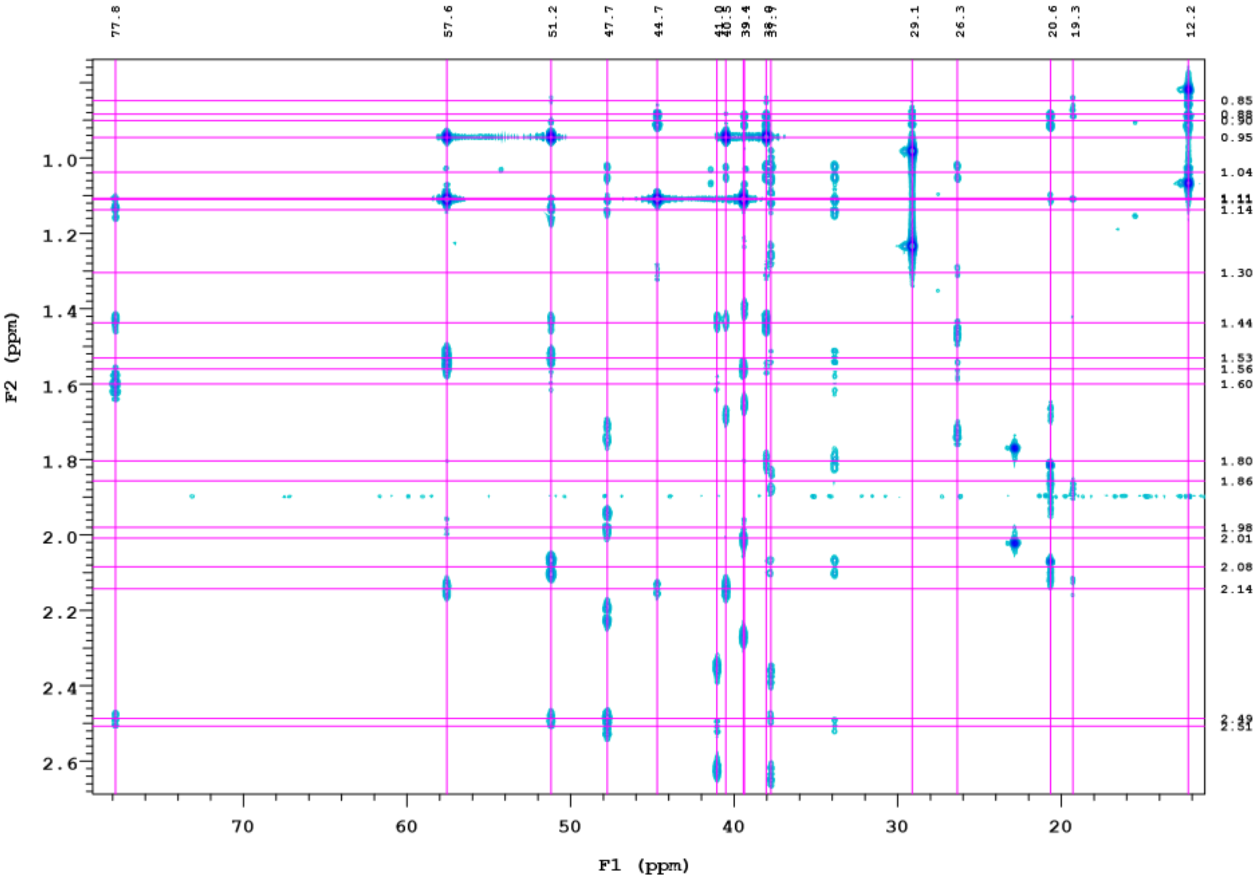

Figure 39. HMBC spectrum of compound 4 from 10 - 80 ppm.

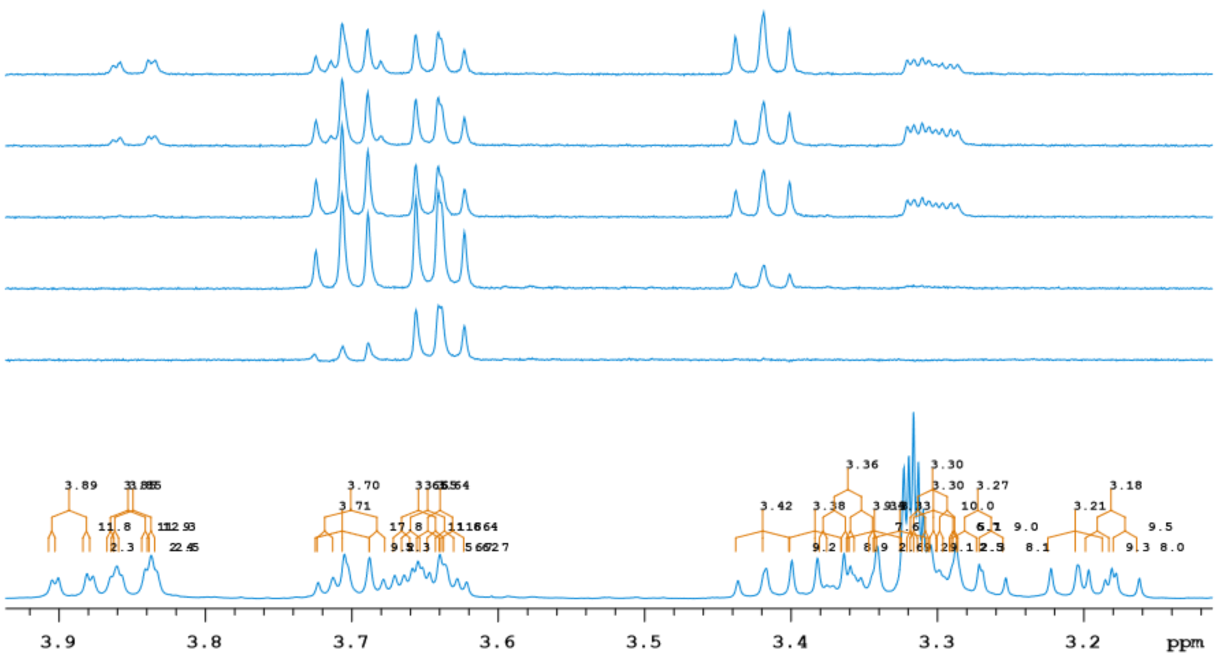

Figure 40: 1H spectrum, bottom, and TOCSY1D spectra with selective excitation at 4.55 ppm, and mixing times from lower to upper, 30, 60, 90, 120 and 150 ms.

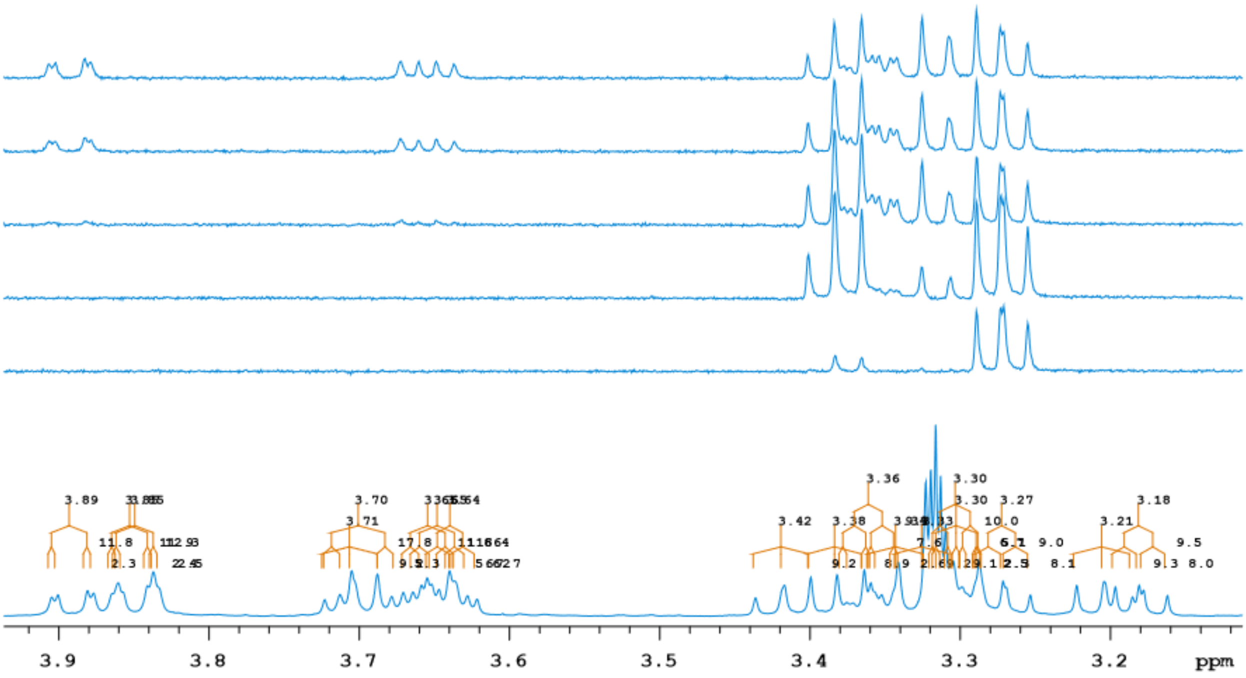

Figure 41: 1H spectrum, bottom, and TOCSY1D spectra with selective excitation at 4.65 ppm, and mixing times from lower to upper, 30, 60, 90, 120 and 150 ms.

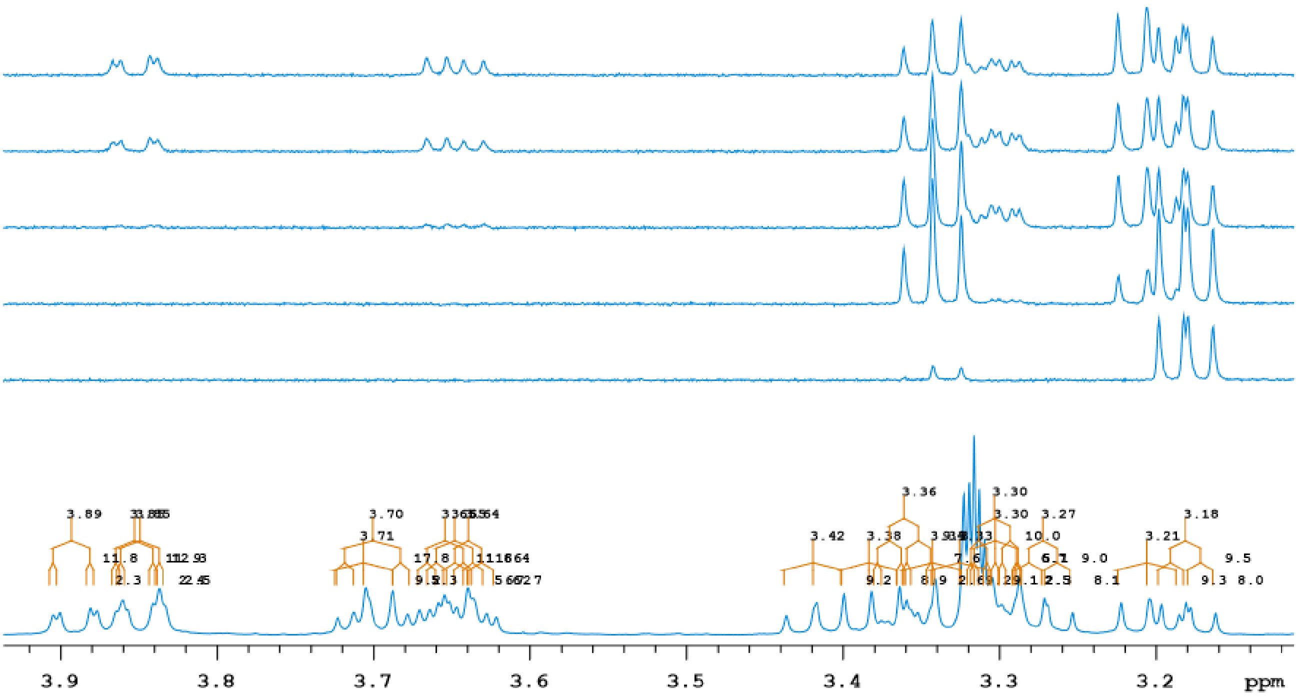

Figure 42: 1H spectrum, bottom, and TOCSY1D spectra with selective excitation at 4.81 ppm, and mixing times from lower to upper, 30, 60, 90, 120 and 150 ms.
